# Blocking matrix metalloproteinase-mediated syndecan-4 shedding restores the endothelial glycocalyx and glomerular filtration barrier function in early diabetic kidney disease

**DOI:** 10.1016/j.kint.2019.09.035

**Published:** 2020-05

**Authors:** Raina D. Ramnath, Matthew J. Butler, Georgina Newman, Sara Desideri, Amy Russell, Abigail C. Lay, Chris R. Neal, Yan Qiu, Sarah Fawaz, Karen L. Onions, Monica Gamez, Michael Crompton, Chris Michie, Natalie Finch, Richard J. Coward, Gavin I. Welsh, Rebecca R. Foster, Simon C. Satchell

**Affiliations:** 1 Bristol Renal, Translational Health Sciences, Bristol Medical School, University of Bristol, Bristol, United Kingdom

**Keywords:** diabetes, glomerular endothelial glycocalyx, matrix metalloproteinase, syndecan-4

## Abstract

The endothelial glycocalyx is a key component of the glomerular filtration barrier. We have shown that matrix metalloproteinase (MMP)-mediated syndecan 4 shedding is a mechanism of glomerular endothelial glycocalyx damage *in vitro*, resulting in increased albumin permeability. Here we sought to determine whether this mechanism is important in early diabetic kidney disease, by studying streptozotocin-induced type 1 diabetes in DBA2/J mice. Diabetic mice were albuminuric, had increased glomerular albumin permeability and endothelial glycocalyx damage. Syndecan 4 mRNA expression was found to be upregulated in isolated glomeruli and in flow cytometry-sorted glomerular endothelial cells. In contrast, glomerular endothelial luminal surface syndecan 4 and Marasmium oreades agglutinin lectin labelling measurements were reduced in the diabetic mice. Similarly, syndecan 4 protein expression was significantly decreased in isolated glomeruli but increased in plasma and urine, suggesting syndecan 4 shedding. *Mmp-2, 9* and *14* mRNA expression were upregulated in isolated glomeruli, suggesting a possible mechanism of glycocalyx damage and albuminuria. We therefore characterised in detail the activity of MMP-2 and 9 and found significant increases in kidney cortex, plasma and urine. Treatment with MMP-2/9 inhibitor I for 21 days, started six weeks after diabetes induction, restored endothelial glycocalyx depth and coverage and attenuated diabetes-induced albuminuria and reduced glomerular albumin permeability. MMP inhibitor treatment significantly attenuated glomerular endothelial and plasma syndecan 4 shedding and inhibited plasma MMP activity. Thus, our studies confirm the importance of MMPs in endothelial glycocalyx damage and albuminuria in early diabetes and demonstrate that this pathway is amenable to therapeutic intervention. Hence, treatments targeted at glycocalyx protection by MMP inhibition may be of benefit in diabetic kidney disease.

## Graphical abstract

**Figure:**
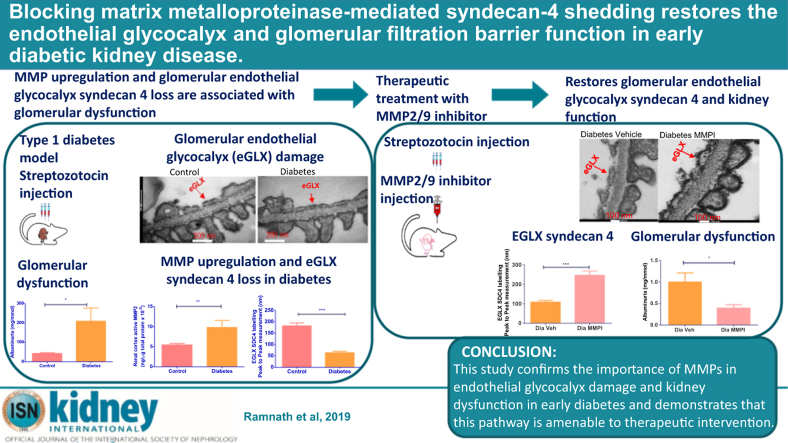

see commentary on page 858

**Translational Statement**

Manipulating glycocalyx, through matrix metalloproteinase (MMP) inhibition, provides an attractive therapeutic target in diabetic kidney disease. MMP inhibitor treatment is realistic in the clinical setting: tetracyclines, antibiotic agents, inhibit MMPs at subantibiotic doses and have been shown to have benefits in human disease,^1^^,^^2^ and low-dose doxycycline (MMP inhibitor) is licensed for the treatment of periodontitis.^3^ The development of more specific MMP inhibitors promises to reduce the adverse effects associated with broad-spectrum MMP inhibitors seen in some clinical trials. Shed glycocalyx components, including syndecan-4, warrant further investigation as potential biomarkers for diabetic kidney disease and associated cardiovascular complications.

Diabetic kidney disease (DKD) is a serious complication of diabetes developing in approximately 1 in 3 people with diabetes. The cost of treating diabetic complications, including DKD, is expected to rise from the current total of £7.7 billion to £13.5 billion by fiscal year 2036 in the United Kingdom.^4^ Despite this, renin-angiotensin system blockade remains the only established treatment in slowing the progression of disease and delaying renal failure. New treatments specifically targeting key steps in disease evolution are desperately needed.

The glomerular endothelium is emerging as a key player in diabetes and other glomerular diseases.^5^ The glomerular endothelial cell (GEC) glycocalyx in particular plays an important role in glomerular barrier function.^6^^,^^7^ The endothelial glycocalyx is a hydrated poly-anionic gel present on the luminal surface of all endothelial cells and is composed principally of proteoglycans.^8^ Proteoglycans consist of a core protein, such as a syndecan (SDC) and glycosaminoglycan side chains, such as heparin sulfate (HS). SDC1 and -4 in particular are prominent in the kidney.^9^, ^10^, ^11^, ^12^ SDC4 has a short cytoplasmic domain and an ectodomain that carries 3 to 5 HS chains^13^, ^14^, ^15^ and is the most abundant among the SDC family in human GEC.^16^

Endothelial glycocalyx has multiple roles in vascular homeostasis,^17^ and its disruption contributes to several vascular diseases including diabetes.^6^, ^7^, ^8^^,^^18^ Our group and others have shown that the glomerular endothelial glycocalyx contributes to the barrier to albumin permeability in cultured cells^18^, ^19^, ^20^ and *in vivo.*^21^, ^22^, ^23^, ^24^ Loss of glycosaminoglycan within the glomerular capillary wall is seen in albuminuric diabetic rats^25^ and diabetic mice.^26^^,^^27^ In humans, systemic glycocalyx dimensions are reduced in diabetes.^28^^,^^29^ Loss of systemic and glomerular glycocalyx is associated with the development of microalbuminuria in diabetes, suggesting that glycocalyx dysfunction contributes to the pathogenesis of this condition.^6^^,^^30^

Glycocalyx components are cleaved from the cell surface by “sheddases” including matrix metalloproteinase (MMPs).^13^^,^^16^ Gelatinases MMP2 and -9 can be activated by membrane type 1 MMP, also known as MMP14. We and others have shown that diabetic conditions induce upregulation of endothelial MMP9,^16^ MMP2,^29^ and urinary MMP14^31^; their enzymatic activities are elevated in diabetic human^31^^,^^32^ and mouse^33^ kidneys. The dysregulation of MMP2 and -9 activities has been associated in the pathophysiology of several diabetic comorbidities.^34^, ^35^, ^36^ Urinary MMP2 and -9 concentration and activity are increased in type 1 diabetic patients with albuminuria.^36^, ^37^, ^38^

We have previously shown that in response to tumor necrosis factor-α (TNF-α), an inflammatory mediator that is increased in diabetic milieu, SDC4 was specifically and significantly upregulated among other SDCs in human GEC *in vitro*.^16^ TNF-α treatment caused a disruption of the GEC glycocalyx through MMP9-mediated shedding of SDC4 and HS, and this was accompanied by an increase in protein permeability across GEC monolayers.^16^ Here we seek to determine whether this mechanism is important in glomerular disease in diabetes *in vivo*. We hypothesize that GEC glycocalyx dysfunction in diabetes is caused by MMP-mediated shedding of SDC4 and that this is amenable to therapeutic intervention.

## Results

### Glomerular endothelial glycocalyx damage accompanies albuminuria in early DKD

**Figure 1:**
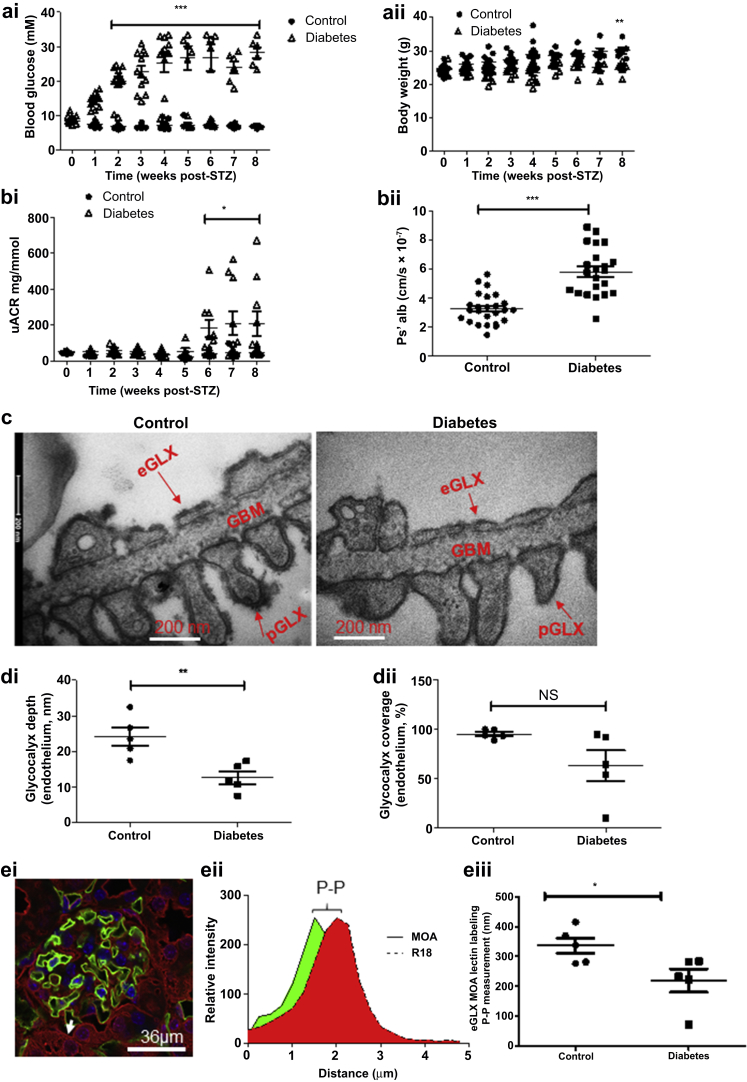
**Glomerular endothelial glycocalyx damage accompanies albuminuria in early diabetic kidney disease.** Streptozotocin (STZ) was given for 5 consecutive days. Two weeks after the first STZ injection, mice became hyperglycemic, which persisted until the mice were killed after week 8 post-STZ. (**ai**) Glycemia at week 2 (control, 6.717 ± 0.1278, *n* = 12 mice; diabetes, 19.78 ± 1.441; *n* = 12 mice; *** *P* < 0.0001) and week 8 post-STZ (control, 6.750 ± 0.1517, *n* = 6 mice; diabetes, 28.22 ± 1.586, *n* = 6 mice; ***P < 0.0001). (**aii**) Body weight, not significant (NS) (*n* = 6 mice each group) when compared with baseline (prior to STZ injection) but significantly lower than control mice at week 8 post-STZ (control, 30.08 ± 0.9420, *n* = 6 mice; diabetes, 24.88 ± 0.7641, *n* = 6 mice; **P = 0.0016). (**bi**) Urinary albumin–creatinine ratio (uACR) was determined post-STZ injection: week 6 (control, 43.94 ± 6.073, *n* = 5 mice; diabetes, 243.1 ± 77.26, *n* = 5 mice; *P = 0.0332), week 7 (control, 43.21 ± 5.644, *n* = 12 mice; diabetes, 209.4 ± 67.88, *n* = 10 mice; *P = 0.0143), and week 8 (control, 42.81 ± 3.352, *n* = 10 mice; diabetes, 208.3 ± 67.74, *n* = 10 mice; *P = 0.0253). (**bii**) Isolated glomeruli from control and diabetic mice were incubated with R18 and then Alexa Fluor 488–bovine serum albumin, and glomerular albumin permeability (Ps’alb) was measured at week 9 post-STZ. Data are presented as follows: control, 3.249 ± 0.2063, *n* = 25 glomeruli; diabetes, 5.806 ± 0.3553, *n* = 22 glomeruli; ***P < 0.0001. The data were also analyzed in terms of mouse number at week 9 post-STZ (control, 3.144 × 10^−7^ ± 2.947 × 10^−8^, *n* = 5; diabetes, 5.857 × 10^−7^ ± 2.788 × 10^−8^, *n* = 5 mice; ***P< 0.0001. (**c**) Representative electron micrographs of the glomerular capillary wall are shown. The measurements were carried out on 3 capillary loops per glomerulus and 2 to 3 glomeruli were used per mouse. Labels indicate endothelial glycocalyx (eGLX), glomerular basement membrane (GBM), and podocyte glycocalyx (pGLX). Bars = 200 nm. Quantification at week 9 post-STZ of (**di**) eGLX depth (control, 24.21 ± 2.550, *n* = 5 mice; diabetes, 12.69 ± 1.755, *n* = 5 mice; **P = 0.0059); (**dii**) percentage endothelium with GLX coverage (control, 95.19 ± 2.139, *n* = 5 mice; diabetes, 63.32 ± 15.35, *n* = 5 mice; NS). (**ei**) Control and diabetic sections were stained with *Marasmium oreades* agglutinin (MOA) lectin and endothelial membrane label R18. Representative image shows glomerular capillaries labeled red (R18) and the luminal endothelial glycocalyx labeled green (MOA). Bar = 36 μm. (**eii,eiii**) Peak-to-peak (P-P) assessment of the glomerular endothelial glycocalyx at week 9 post-STZ showed a significant reduction in glycocalyx thickness in diabetes (control, 336.0 ± 26.33, *n* = 5 mice; diabetes, 219.0 ± 38.82, *n* = 5 mice; *P = 0.04). Each dot, triangle, and square on the graph represents a mouse. Data are expressed as the mean ± SEM, and unpaired Student *t* test was used for statistical analysis. To optimize viewing of this image, please see the online version of this article at www.kidney-international.org.

DBA/2J mice became hyperglycemic 2 weeks after the first of 5 daily streptozotocin STZ injections (Figure 1ai). There was no significant change in body weight in the STZ-treated mice when compared with baseline, prior to STZ injection (Figure 1aii). However, the diabetic mice gained less weight than control mice did, resulting in a 1.2-fold lower body weight after week 8 post-STZ (Figure 1aii). The mice became significantly albuminuric after week 6 post-STZ, and this persisted until the mice were killed with a 4.9-fold increase in albuminuria (Figure 1bi) at 8 week post-STZ. Compromised glomerular capillary wall integrity was confirmed by a 2-fold increase in glomerular albumin permeability (Ps’alb) (Figure 1bii). Permeability changes were associated with disruption of the glomerular glycocalyx. There was a 2-fold decrease in endothelial glycocalyx depth measured by electron microscopy but no significant change in glycocalyx coverage (Figure 1c and d). Moreover, a decrease in podocyte glycocalyx depth was observed, but there was no significant change in other ultrastructural features, including glomerular basement membrane (GBM) thickness, slit diaphragm, and foot process width (Supplementary Figure S1A–E). Moreover, picrosirius red staining showed no significant collagen deposition in diabetes when compared with control (Supplementary Figure S1F and G), confirming the electron microscopy results, suggesting no change in GBM structure. *Marasmium oreades* agglutinin (MOA) lectin bound specifically to the endothelial glycocalyx, on the luminal surface of the GEC (determined using the membrane label R18) (Figure 1ei). We have applied our peak-to-peak measurement technique, previously used *in vivo*,^39^^,^^40^ for the first time on fixed kidney tissue (Figure 1eii). Peak-to-peak measurement of MOA labeling, an index of glycocalyx thickness, demonstrated a 1.5-fold reduction in endothelial glycocalyx thickness in diabetes that is consistent with the reduction in endothelial glycocalyx depth observed by electron microscopy (Figure 1eiii).

### Glycocalyx-related gene expression is modified in early DKD

**Figure 2:**
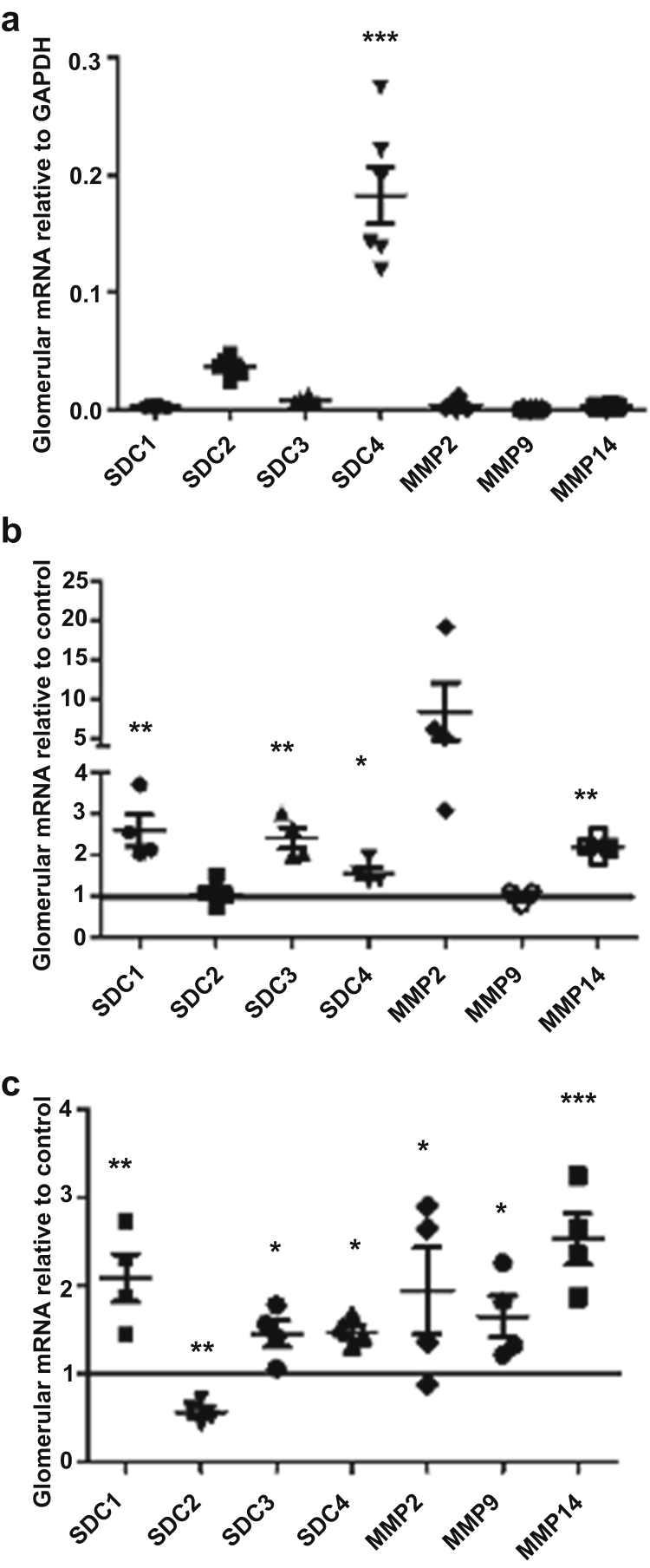
**Glycocalyx-related gene expression is modified in early diabetic kidney disease.** (**a**) Glomerular mRNA expression of syndecans (*Sdcs*) and matrix metalloproteinases (*Mmps*) relative to glyceraldehyde-3-phosphate dehydrogenase (GAPDH) from control mice was determined. ***P < 0.0005, 1-way analysis of variance with Bonferroni as a *post hoc* test at week 8 post-streptozotocin (STZ); *n* = 6 mice for all the genes except *n* = 4 mice for MMP14. (**b**) A custom-designed TaqMan quantitative polymerase chain reaction array was used to identify changes in glycocalyx-related gene expression in diabetic kidney disease. The 2^−ΔΔCT^ method of quantification was used to calculate the fold change relative to control; *P < 0.05, **P < 0.005; unpaired Student *t* test (control, *n* = 6 mice, and diabetes, *n* = 4 mice, for all the genes; except control, *n* = 4 mice, and diabetes, *n* = 4 mice, for MMP14; and diabetes, *n* = 3 mice, for MMP9). (**c**) Genes of interest were validated using independent quantitative polymerase chain reaction (control, *n* = 6 mice; diabetes, *n* = 4 mice); *P < 0.05, **P < 0.005, ***P < 0.0005; unpaired *t* test at week 8 post-STZ. Each dot, square, or triangle on the graph represents a mouse.

Expression of the glomerular glycocalyx-related genes *Sdcs* and glycocalyx modifying genes *Mmp*s relative to glyceraldehyde-3-phosphate dehydrogenase in untreated mice was determined. *Sdc44* mRNA expression was significantly higher than other *Sdc* and *Mmp* mRNAs (Figure 2A), in line with *Sdc* expression in human GEC.^16^ The effect of diabetes on the expression of different components of the glycocalyx was investigated in isolated glomeruli using a custom-designed TaqMan quantitative polymerase chain reaction (qPCR) array focused on glycocalyx-related genes (Figure 2b, Supplementary Table S1) similar to that used previously.^16^ Expression Suite software (Thermo Fisher Scientific, Waltham, MA) and 2^−ΔΔ*CT*^ method followed by Student *t* test were used to analyze the array data and both methods showed a significant increase in *Sdc1*, *Sdc4*, and *Mmp14* mRNA expression among other significantly modulated gene expressions (Figure 2b, Supplementary Table S2). *Sdc1*, *-3*, and *-4*, and *Mmp14* were independently validated by real-time qPCR, demonstrating significant upregulation in all of them (Figure 2c). Although there was no significant increase in *Mmp2* and *-9* mRNA expression with the TaqMan qPCR array, because of previous data suggesting their importance they were further examined by individual qPCR, which showed significant increase in *Mmp2* and *-9* (Figure 2c). In contrast, *Sdc2* was significantly downregulated (Figure 2c).

### *Sdc4* mRNA, protein, and MMP activity are altered in early DKD

**Figure 3:**
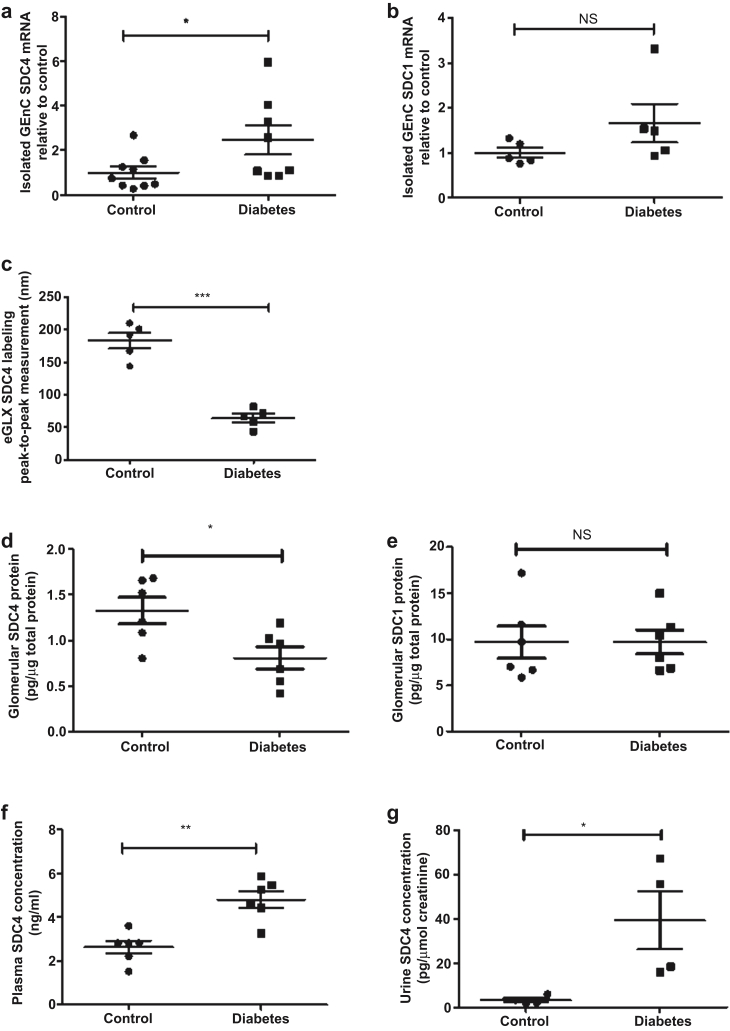
**Syndecan-4 (*Sdc4*) mRNA and protein are altered in early diabetic kidney disease (DKD).** Glomerular endothelial cells (GECs) were sorted by fluorescence-activated cell sorting, and (**a**) *Sdc4* and (**b**) *Sdc1* mRNA expression were determined. The 2^−ΔΔCT^ method of quantification was used to calculate the fold change, normalized to glyceraldehyde-3-phosphate dehydrogenase, at week 8 post-streptozotocin (STZ), (*Sdc4*: control, 1.000 ± 0.2565, *n* = 9 mice; diabetes, 2.470 ± 0.6580, *n* = 8 mice; *P < 0.05; *Sdc1*: control, 1.000 ± 0.1112, *n* = 5 mice; diabetes, 1.663 ± 0.4289, *n* = 5 mice; nonsignificant [NS]). Control and diabetic kidney sections were labeled with SDC4 and endothelial membrane label R18. (**c**) Peak-to-peak assessment of the glomerular endothelial glycocalyx using SDC4 labeling at week 9 post-STZ showed a significant reduction in glycocalyx SDC4 in diabetes (control, 182.5 ± 12.04, *n* = 5 mice; diabetes, 64.31 ± 6.513, *n* = 5 mice; ***P< 0.0001). Isolated glomerular lysates from control and diabetic mice were used to determine (**d**) SDC4 and (**e**) SDC1 concentration using SDC4 and SDC1 ectodomain enzyme-linked immunosorbent assay (ELISA). The data were then normalized to total protein content. SDC4 (control, 1.327 ± 0.1439, *n* = 6 mice; diabetes, 0.8059 ± 0.1218, *n* = 6 mice; *P < 0.05); SDC1 (control, 9.671 ± 1.742, *n* = 6 mice; diabetes, 9.699 ± 1.313, *n* = 6 mice; NS) at week 8 post-STZ. SDC4 shedding in the (**f**) plasma and (**g**) urine was determined in DKD using the SDC4 ectodomain ELISA at week 8 post-STZ. For plasma: control, 2.631 ± 0.2860, *n* = 6 mice; diabetes, 4.801 ± 0.3793, *n* = 6 mice; **P < 0.005. Urine SDC4 was normalized to creatinine (control, 3.343 ± 0.9410, *n* = 4 mice; diabetes, 39.32 ± 13.03, *n* = 4 mice; *P < 0.05; Mann-Whitney test at week 8 post-STZ). Each dot or square on the graph represents a mouse. Data are expressed as the mean ± SEM, and unpaired Student *t* test was used for statistical analysis unless specified.

**Figure 4:**
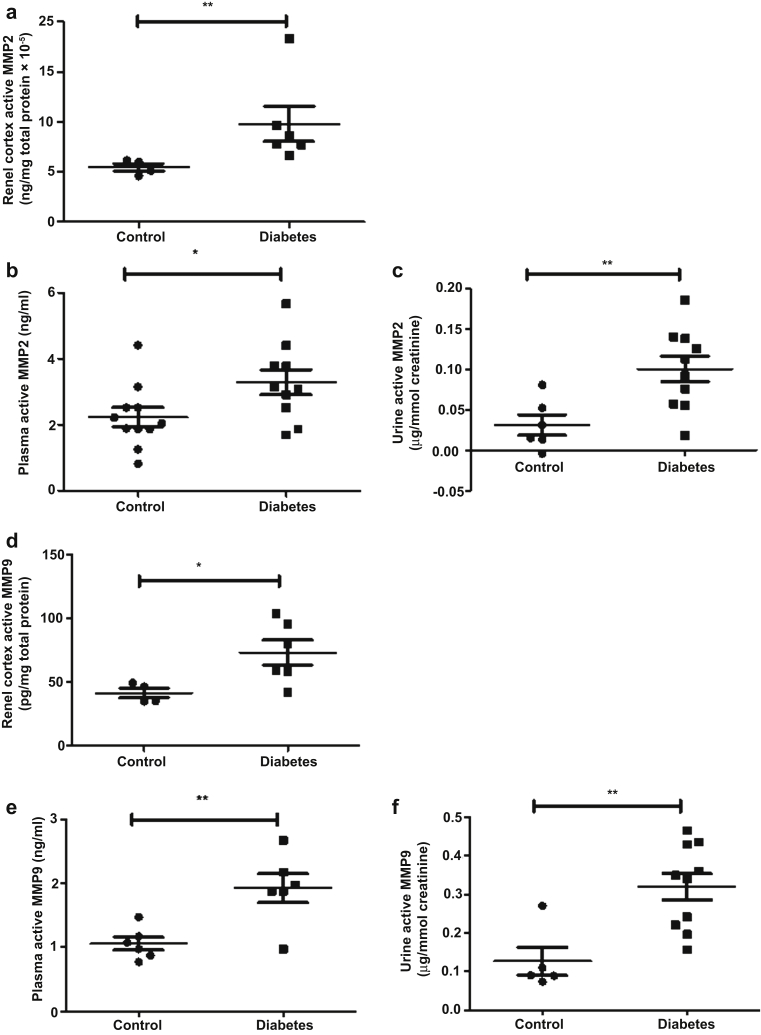
**Matrix metalloproteinase (MMP) activity is altered in multiple compartments in early diabetic kidney disease (DKD).** Renal cortex lysates from control and diabetic mice were used to determine (**a**) MMP2 (control, 5.462 ± 0.3652, *n* = 4 mice; diabetes, 9.797 ± 1.753, *n* = 6 mice) and (**d**) MMP9 (control, 41.34 ± 3.85, *n* = 4 mice; diabetes, 73.12 ± 9.85, *n* = 6 mice) activities using MMP2 and MMP9 Biotrak Activity Assays (GE Healthcare Life Sciences, Buckinghamshire, UK). The data were then normalized to total protein content at week 8 post-streptozotocin (STZ); **P = 0.0095, Mann-Whitney test, and **P = 0.037, unpaired Student *t* test were carried out, respectively. Systemic circulation of (**b**) plasma MMP2 (control, 2.232 ± 0.2873, *n* = 11 mice; diabetes, 3.285 ± 0.3760, *n* = 10 mice; *P = 0.0365), (**c**) urine MMP2 (control, 0.03142 ± 0.01251, *n* = 6 mice; diabetes, 0.1002 ± 0.01577, *n* = 10 mice; **P = 0.0091), (**e**) plasma MMP9 (control, 1.053 ± 0.1014, *n* = 6 mice; diabetes, 1.920 ± 0.2262, *n* = 6 mice; **P = 0.0058), and (**f**) urine MMP9 (control, 0.1264 ± 0.03644, *n* = 5; diabetes, 0.3196 ± 0.03456, *n* = 10 mice; **P = 0.0080 Mann-Whitney test) activities were determined in DKD. Urine MMP2 and MMP9 were normalized to urine creatinine at week 8 post-STZ. Each dot or square on the graph represents a mouse. Data are expressed as the mean ± SEM, and unpaired *t* test was used for statistical analysis unless specified.

Expression of *Sdc4* and *-1* mRNA specifically in GEC was determined in cells isolated by flow cytometry (Supplementary Figure S2). *Sdc4* mRNA expression was upregulated 2.5-fold (Figure 3a), whereas *Sdc1* mRNA expression was not significantly changed in diabetes (Figure 3b). To quantify endothelial glycocalyx SDC4 protein expression, an anti-SDC4 antibody was injected intravenously and shown to localize predominantly to GEC (Supplementary Figure S3). To specifically quantify the SDC4 on the luminal surface of the endothelial cells, we have used R18 labeling to highlight the endothelial membrane and enable separation of luminal from nonluminal labeling. Peak-to-peak measurement demonstrated a 2.8-fold reduction in glomerular endothelial glycocalyx SDC4 in diabetes (Figure 3c). Similarly, SDC4 expression by enzyme-linked immunosorbent assay (ELISA) was reduced 1.6-fold in diabetic glomeruli, suggesting glomerular SDC4 shedding (Figure 3d), whereas SDC1 glomerular protein expression was not changed in diabetes (Figure 3e). There was a 1.8-fold (Figure 3f) and an 11.8-fold (Figure 3g) increase in plasma and urine SDC4 concentrations, respectively, again consistent with systemic SDC4 shedding in diabetes. We measured the sheddases MMP2 and -9 and found that active MMP2 concentration was increased by 2-fold in kidney cortex lysate, 1.5-fold in plasma, and 3-fold in urine (Figure 4a–c) and active MMP9 concentration was increased by 1.8-fold in kidney cortex lysate, 1.8-fold in plasma, and 2.5-fold in urine (Figure 4d–f).

### MMP2 and -9 mediate disruption of the glomerular endothelial glycocalyx in early DKD

**Figure 5:**
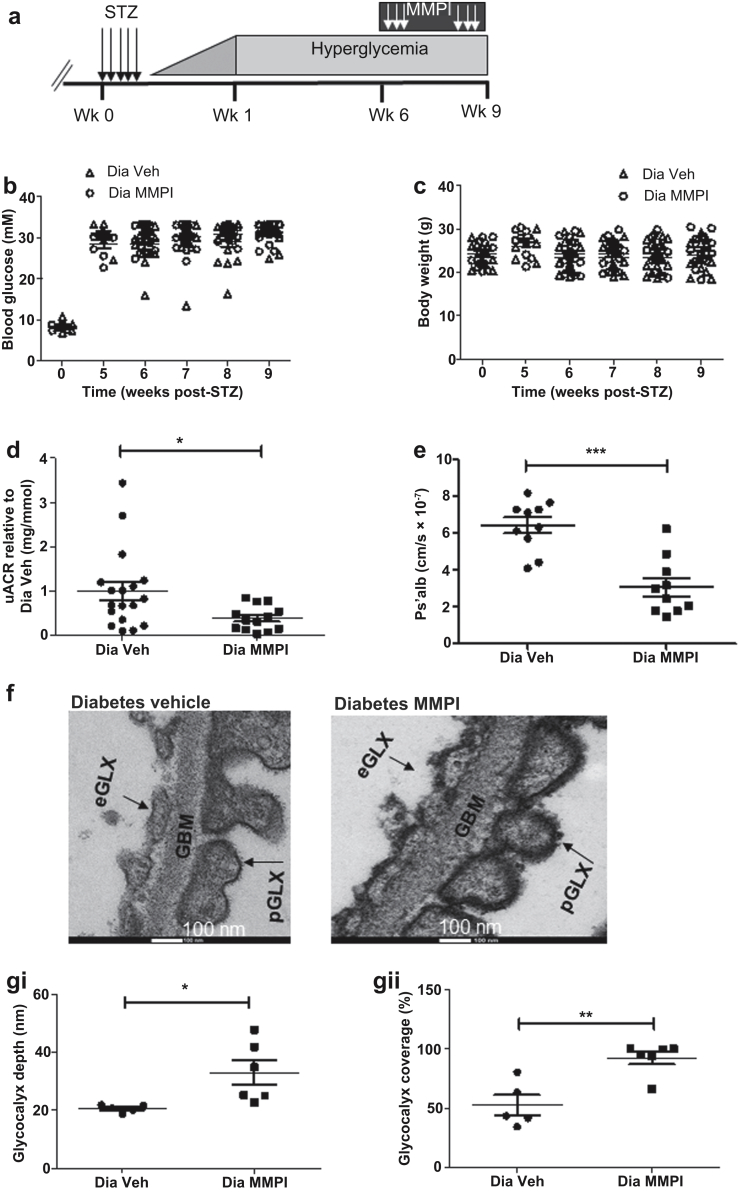
**Matrix metalloproteinases (MMPs) mediate disruption of the glomerular endothelial glycocalyx in early diabetic kidney disease.** (**a**) Schematic overview of the procedure; 5 daily injections of streptozotocin (STZ) were given at week 0. Six weeks post-STZ injection, MMP2 and MMP9 inhibitor (MMPI) was given for 21 days and mice were killed at week 9 post-STZ injection. MMPI had no significant effect on (**b**) glycemia (diabetic [Dia] vehicle [Veh], 31.43 ± 0.8017, *n* = 13 mice; Dia MMPI, 31.09 ± 0.6403, *n* = 14 mice; nonsignificant) and (**c**) body weight (Dia Veh, 23.88 ± 1.044, *n* = 13 mice; Dia MMPI, 24.83 ± 0.9469, *n* = 14 mice, nonsignificant). (**d**) Treatment with MMPI reduced urine albumin–creatinine ratio (uACR) (Dia Veh, 1.000 ± 0.2093, *n* = 18 mice; Dia MMPI, 0.3925 ± 0.077, *n* = 13 mice; *P = 0.0172, Mann-Whitney test at week 9 post-STZ). The fold change of diabetic MMPI relative to diabetic vehicle was calculated to enable pooling of results from different experiments. (**e**) Isolated glomeruli from diabetic + MMPI or vehicle mice were incubated with R18 and then Alexa Fluor 488–bovine serum albumin, and glomerular albumin permeability (Ps’alb) was measured. Data are presented as follows: Dia Veh, 6.438 ± 0.4302, *n* = 10 glomeruli; Dia MMPI, 3.081 ± 0.4858, *n* = 10 glomeruli; ***P < 0.0001. The data were also analyzed in terms of mouse number (Dia Veh, 6.533 × 10^−7^ ± 2.450 × 10^−8^, *n* = 3; 3.035 × 10^−7^ ± 2.963 × 10^−8^, *n* = 3 mice; ***P = 0.0008). (**f**) Diabetic + MMPI or vehicle mice were perfusion-fixed for electron microscopy with cacodylate buffer containing glutaraldehyde and Alcian blue. Representative electron micrographs of the glomerular capillary wall are shown. The measurements were carried out on 3 capillary loops per glomerulus, and 2 to 3 glomeruli were used per mouse. Labels indicate endothelial glycocalyx (eGLX), glomerular basement membrane (GBM), and podocyte glycocalyx (pGLX). Bars = 100 nm. Quantification of (**gi**) eGLX depth (Dia Veh, 20.69 ± 0.5772, *n* = 5 mice; Dia MMPI, 32.91 ± 4.186, *n* = 6 mice; *P = 0.0276). (**gii**) Percentage of endothelium with GLX coverage (Dia Veh, 52.74 ± 8.411, n = 5 mice; Dia MMPI, 92.56 ± 5.383, *n* = 6 mice; **P = 0.0026). Each dot or square on the graph represents a mouse. Data are expressed as the mean ± SEM, and unpaired Student *t* test at week 9 post-STZ was used for statistical analysis unless specified. To optimize viewing of this image, please see the online version of this article at www.kidney-international.org.

An MMP2 and -9 inhibitor (MMPI) was given therapeutically, after the onset of albuminuria at week 6 post-STZ, for 21 days (Figure 5a). Blockade of MMP had no significant effect on glycemia and body weight (Figure 5b and c) but significantly attenuated the diabetes-induced increase in urinary albumin creatinine ratio by 2.6-fold (Figure 5d and Supplementary Table S4). MMPI treatment reduced the diabetes-mediated increase in Ps’alb by 2-fold, restoring the permeability barrier of the glomerular capillaries (Figure 5e). MMPI also restored diabetes-induced endothelial glycocalyx loss, evidenced by a 1.6-fold increase in endothelial glycocalyx depth and a 2-fold increase in endothelial glycocalyx coverage (Figure 5f and g). MMPI treatment also resulted in a significant increase in podocyte glycocalyx depth, but there was no significant effect on GBM thickness, nor podocyte foot process and slit diaphragm width (Supplementary Figure S4A–E).

### Blockade of MMP2 and -9 ameliorates diabetes-induced changes in SDC4 and reduces plasma MMP activity

**Figure 6:**
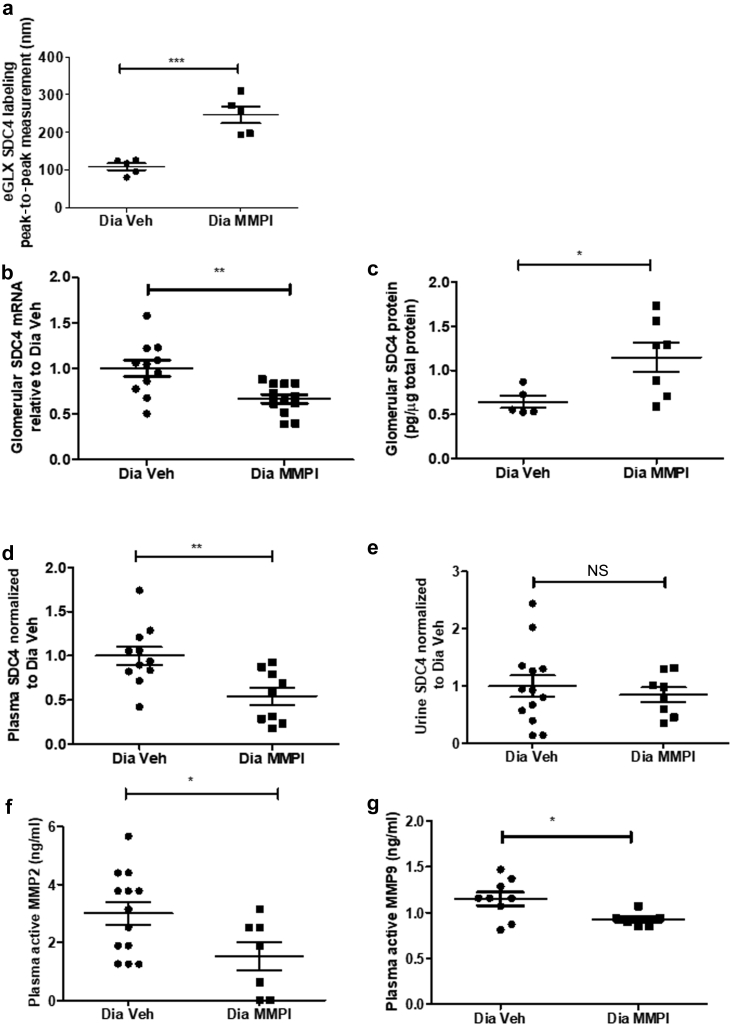
**Blockade of matrix metalloproteinase-2 (MMP2) and matrix metalloproteinase-9 (MMP9) ameliorates diabetes-induced changes in syndecan-4 (SDC4) and reduces plasma MMP activity.** Diabetic (Dia) + MMP2 and MMP9 inhibitor (MMPI) or vehicle (Veh)-treated kidney sections were labeled with SDC4 and endothelial membrane label R18. (**a**) Peak-to-peak assessment of the glomerular endothelial glycocalyx (eGLX) using SDC4 labeling at week 9 post-streptozotocin showed a significant restoration in glycocalyx thickness (Dia Veh, 109.2 ± 9.003, *n* = 5 mice; Dia MMPI, 246.0 ± 22.36, *n* = 5 mice; ***P = 0.0005). (**b**) Isolated glomerular lysate from diabetic + MMPI or vehicle-treated mice was used to determine *Sdc4* mRNA expression. The 2^−ΔΔCT^ method of quantification was used to calculate the fold change, normalized to glyceraldehyde-3-phosphate dehydrogenase and relative to Dia Veh (Dia Veh, 1.000 ± 0.089, *n* = 11 mice; Dia MMPI, 0.6658 ± 0.04849, *n* = 12 mice; **P = 0.0029). (**c**) Isolated glomerular lysate from diabetic + MMPI or vehicle-treated mice was used to determine SDC4 concentration with previously used SDC4 ectodomain enzyme-linked immunosorbent assay. The data were then normalized to total protein content (Dia Veh, 0.6426 ± 0.06780, *n* = 5 mice; Dia MMPI, 1.150 ± 0.1634, *n* = 7 mice; *P = 0.0322). MMPI attenuated SDC4 shedding in the (**d**) plasma (Dia Veh, 1.000 ± 0.1029, *n* = 11 mice; Dia MMPI, 0.5449 ± 0.09749, *n* = 9 mice; **P = 0.0054), but not in the (**e**) urine (Dia Veh, 1.000 ± 0.1895, *n* = 13 mice; Dia MMPI, 0.8501 ± 0.1290, *n* = 8 mice; nonsignificant [NS]) in diabetic kidney disease. The fold change of diabetic MMPI relative to diabetic vehicle was calculated to enable pooling of results from different experiments. (**f,g**) Plasma MMP2 (Dia Veh, 3.001 ± 0.3977, *n* = 13 mice; Dia MMPI, 1.528 ± 0.4924, *n* = 7 mice; *P = 0.0366) and MMP9 (Dia Veh, 1.150 ± 0.07157, *n* = 9 mice; Dia MMPI, 0.9266 ± 0.03281, *n* = 6 mice; *P = 0.0314) activities, using MMP2 and MMP9 Biotrak Activity Assays (GE Healthcare Life Sciences, Buckinghamshire, UK), were reduced in the diabetic + MMPI group when compared with the diabetic + vehicle group. Each dot or square on the graph represents a mouse. Data are expressed as the mean ± SEM, and unpaired Student *t* test at week 9 post-streptozotocin was used for statistical analysis unless specified.

MMPI treatment restored the decrease in SDC4 labeling by peak-to-peak measurement 2.2-fold (Figure 6a). It attenuated the increase in *Sdc4* mRNA expression observed in diabetes by 1.5-fold (Figure 6b) and restored the loss of glomerular SDC4 protein expression by 1.8-fold (Figure 6c). MMPI significantly attenuated diabetes-induced plasma SDC4 shedding by 2-fold but not urine SDC4 shedding (Figure 6d and e). Therapeutic treatment with MMPI blocked diabetes-induced increase in plasma MMP2 and -9 activities by 2- and 1.2-fold, respectively (Figure 6f and g), confirming that this inhibitor reduces the activity of both MMPs *in vivo*.

## Discussion

In this study, we investigated our hypothesis that GEC glycocalyx dysfunction in diabetes is caused by MMP-mediated SDC4 shedding, and importantly, that it is amenable to therapeutic intervention. We have used a model of early DKD, weeks 8 to 9 post-STZ, resulting in the mice being hyperglycemic and albuminuric. Due to tubular reabsorption of filtered albumin and changes in local hemodynamics, albuminuria is not a sensitive measure of glomerular permeability. Therefore, we have used our reliable, physiologically relevant glomerular Ps’alb assay to directly measure glomerular permeability using single capillaries in isolated glomeruli. The assay has been extensively validated and provided robust, reproducible estimates of glomerular Ps’alb.^24^ We observed an increase in glomerular Ps’alb, confirming reduced glomerular capillary wall integrity in early DKD. The increase in albuminuria and in glomerular permeability was associated with a reduction in endothelial and podocyte glycocalyx depth without affecting other glomerular ultrastructures such as GBM thickness, podocyte slit diaphragm, and foot process width. Moreover, there was no change in collagen deposition in our model using picrosirius red staining, and this supports the electron microscopy data. Our findings are consistent with our previous work showing that at 8 weeks post-STZ injection, type 1 diabetic mice do not develop GBM thickness and podocyte effacement.^41^ GBM thickening has been reported in some but not all mouse models of diabetic nephropathy.^42^ For example, GBM thickening occurs in diabetic DBA2J mice after ∼25 weeks of hyperglycemia.^43^ Our data confirm previous observations that loss of glomerular glycocalyx, in the absence of changes in other glomerular capillary wall components, is associated with albuminuria and glomerular permeability in early DKD.^24^^,^^27^

The role of the podocyte glycocalyx has not previously been examined in detail so further work is necessary to determine the significance of our observation of podocyte glycocalyx loss in diabetes. Some studies support the hypothesis that endothelial activation can lead to secondary podocyte injury.^44^, ^45^, ^46^, ^47^ It has been shown that damage specifically to the glomerular endothelial glycocalyx using hyaluronidase results in extensive uptake of albumin by podocytes, which could initiate an inflammatory reaction resulting in podocyte damage.^48^^,^^49^ In this study, we observed endothelial and podocyte glycocalyx damage, but the damage to podocytes was not severe enough to cause foot process effacement in this model of early DKD.

MOA lectin, known to bind to specific carbohydrate sequences present in the glycocalyx of mouse glomerular endothelium,^50^^,^^51^ was used to specifically study the glomerular endothelial glycocalyx. Consistent with previous findings,^50^^,^^51^ MOA lectin localized to the glomerular endothelial glycocalyx, and our peak-to-peak measurement technique,^39^^,^^40^ demonstrated a reduction in endothelial glycocalyx thickness in diabetes, which complements and supports the electron microscopy data. The peak-to-peak measurement using MOA lectin labeling is greater than the glycocalyx thickness by electron microscopy. This is likely to be due to endothelial glycocalyx collapse with the dehydration procedure that follows fixation in preparation for electron microscopy.^52^

The glycocalyx is a complex structure, and which components are damaged in diabetes has not been previously defined. We show here that in untreated control mice, *Sdc4* mRNA was highly expressed in isolated glomeruli, which is in line with the *Sdc4* mRNA expression in human conditionally immortalized GEC.^16^ A custom-designed qPCR array, focusing on glycocalyx-related genes, was used to determine the effect of diabetes on glomerular glycocalyx components. Proteoglycan *Sdc1* and *-4* mRNA expression were considerably upregulated in isolated glomeruli in DKD. This is consistent with another study showing a 26-fold increase in renal or glomerular, or both, *Sdc4* mRNA in proteinuric DKD.^53^ Furthermore, by using fluorescence-activated cell-sorted mouse GEC, we were able to show that *Sdc4* mRNA expression was specifically upregulated in this cell type. Our previous work on cultured human GEC indicate that this *Sdc4* mRNA upregulation is a compensatory response to SDC4 shedding.^16^ Indeed in line with the MOA lectin results, we showed a reduction in glomerular endothelial SDC4 peak-to-peak measurement in DKD. Similarly, glomerular glycocalyx SDC4 protein expression was considerably reduced in DKD, suggesting shedding of glomerular endothelial glycocalyx SDC4. This was also accompanied by an increase in plasma and urine SDC4, suggesting systemic glycocalyx shedding. Other glycocalyx components, including SDC1 concentration, have already been reported to be increased in serum from subjects with type 1 diabetes mellitus and microalbuminuria.^54^ SDC4 is unlikely to be the only constituent that is being shed from the glycocalyx in diabetes. SDC4 carries HS side chains so HS shedding is likely to accompany SDC4 shedding as we have shown previously *in vitro*^16^ and others have shown *in vivo* in a diabetes model.^55^ Glycocalyx component hyaluronan^56^ has also been shown to be shed in diabetes.

We then determined the underlying mechanism of SDC4 shedding in DKD. MMP2 and -9 are known to increase in human type 1 and 2 diabetes^57^, ^58^, ^59^, ^60^ and their levels are altered in animal DKD models.^31^^,^^32^^,^^38^^,^^60^ In this study, we characterized in detail expression of relevant sheddases in different compartments and showed that *Mmp2* and *-9* were upregulated at the mRNA level and their activities were enhanced in kidney cortex, plasma, and urine in DKD. We also observed an increase in *Mmp14* mRNA expression in DKD, which was not surprising considering MMP14 is a known gelatinase activator.^61^ The increase in MMP activity was associated with loss in GEC glycocalyx and an increase in albuminuria, suggesting that MMP could be the underlying mechanism in SDC4 ectodomain shedding. It is likely that MMPs have other targets and that SDC4 loss is among them. Indeed, MMPs mediate degradation of other proteoglycans,^62^ SDC1,^63^ tight junction protein ZO-1,^64^ and the antioxidant enzyme superoxide dismutase.^63^ MMPs may also degrade collagens, but we did not find any evidence of this in our model of early DKD. Published findings support that MMPs mediate degradation of endothelial glycocalyx^65^, ^66^, ^67^, ^68^ and are consistent with published work highlighting MMP2 and -9 cleavage sites on the SDC4 ectodomain.^69^

To determine whether endothelial glycocalyx could be protected or restored in DKD, a pharmacological approach was adopted, and MMPI, a highly selective inhibitor for MMP2 and -9^70^ was given therapeutically after the onset of albuminuria. MMPI had no effect on blood glucose or body weight, but it significantly attenuated the increase in albuminuria. Our glomerular permeability assay confirmed that MMPI reduced the increase in albuminuria by decreasing glomerular Ps’alb in DKD, highlighting the usefulness of our novel assay in detecting benefits of treatments in terms of glomerular permeability. A study carried out in MMP9-deficient mice has shown significant attenuation of albuminuria and hyperfiltration in diabetic nephropathy but without determining its effects on the glycocalyx.^33^ We have shown that MMPI treatment protected endothelial glycocalyx by significantly increasing glycocalyx depth and coverage. The inhibitor had no effect on glomerular ultrastructure including GBM thickness, podocyte foot process, and slit diaphragm width. This was as expected given that we did not observe changes in these parameters in our model of early DKD. These data are consistent with our previously published work that treatment with vascular endothelial growth factor A_165b_ and angiopoietin-1 reversed damage to the glomerular endothelial glycocalyx and normalized glomerular permeability in early DKD.^24^^,^^27^ Moreover, vascular endothelial growth factor C also protected against raised glomerular permeability and ameliorated glycocalyx disruption in diabetic glomeruli.^41^

Blockade of MMP2 and -9 attenuated diabetes-induced changes in glomerular endothelial glycocalyx *Sdc4* and glomerular SDC4 at the mRNA and protein levels, suggesting a reduction in glomerular endothelial SDC4 shedding. We previously observed similar effects in human GEC, where MMP inhibition attenuated *Sdc4* mRNA synthesis and SDC4 shedding, resulting in a reduction in Ps’alb.^16^ MMPI attenuated diabetes-induced SDC4 shedding in the plasma but not urine. Our data show that MMPI has reached the podocytes as it restored the podocyte glycocalyx loss observed in diabetes. So, this suggests that there are potentially different mechanisms contributing to SDC4 shedding in the plasma and urine; the latter being resistant to MMPI, perhaps other proteases might be responsible for SDC4 shedding in the urine. Future work will determine the relative importance of MMP2 or -9 and their potential cellular sources. We have previously shown *in vitro* that MMPs produced by GEC are sufficient to cause SDC4 shedding,^16^ but circulating immune cells also produce MMPs,^71^^,^^72^ which may be equally or more important in diabetes *in vivo*. There a number of upstream signaling pathways (MAP kinases,^73^^,^^74^ nuclear factor κB, and protein kinase C^65^) that have been implicated in MMP activation, but there is little context-specific information, and further work in this area may identify further targets for therapeutic intervention. For example, we have previously shown that TNF-α activates MMPs and TNF inhibitor used in clinical practice (etanercept) attenuates glycocalyx loss in an experimental endotoxin model.^75^

Disruption of the endothelial glycocalyx is known to increase albumin excretion *in vivo*^23^ and *in vitro*.^16^^,^^20^ Endothelial glycocalyx protection or restoration is therefore an attractive therapeutic target not least because the endothelial glycocalyx damage occurs early in DKD when microalbuminuria is present but glomerular ultrastructure is otherwise unaffected, as we show here. Treatment at this stage may prevent downstream consequences of developing albuminuria. We have demonstrated that MMP2 and -9–mediated SDC4 shedding is the underlying mechanism of endothelial glycocalyx damage in DKD and that this pathway is potentially amenable to therapeutic intervention.

## Methods

### Animal welfare

All animal experiments were approved by the UK Home Office. Male DBA/2J, 6-week-old mice (20–25 g), purchased from Charles River UK Limited (Kent, UK) were maintained in an environment with controlled temperature (21–24 °C) and lighting (12:12 hour light–dark cycle). Standard laboratory chow and drinking water were provided *ad libitum*. A period of 1 to 2 weeks was allowed for animals to acclimatize before any experiments.

### Type 1 diabetes model

For induction of diabetes, mice were fasted for 4 to 6 hours before using the low-dose Diabetic Complications Consortium protocol.^42^ Briefly, STZ was given i.p. at 50 mg/kg for 5 consecutive days and the mice were killed at 8 or 9 weeks post-STZ injection.S6 Glycemia, by tail-tip blood droplet analysis using a glucometer (Accu-Chek Aviva; Roche, Basel, Switzerland) and body weight were monitored weekly after STZ. STZ-injected mice with glycemia ≥15 mmol/l were considered diabetic and included in the study. The mice were placed in metabolic cages for up to 4 hours to collect urine weekly. Urinary albumin was quantified with a mouse albumin ELISA (Bethyl Laboratories, Inc., Montgomery, TX), and creatinine was measured using an enzymatic spectrophotometric assay (Konelab T-Series 981845; Thermo Fisher Scientific, Vantaa, Finland). Urinary albumin-to-creatinine ratio was calculated as previously described.^27^ At 6 weeks after STZ administration, some mice were given daily i.p. injections of a potent and highly selective MMPI biphenylylsulfonylamino-3-phenylpropionic acid^40^^,^^70^^,^^76^^,^^77^ (444241; Merck, Middlesex, UK) at 5 mg/kg or vehicle (0.05% dimethylsulfoxide in phosphate-buffered saline [PBS]) for 21 days and the mice were killed at 9 weeks. MMPI binds to the zinc ion at the active site of MMP2 and -9, thereby blocking their activities.^78^

For more details on MMPI and tissue collection in type 1 diabetes model, please see the Supplementary Methods.

### Glomerular Ps’alb assay

The glomerular Ps’alb assay was carried out as previously described.^24^ Briefly, Ringer-perfused kidney was sieved in 4% bovine serum albumin (BSA) in Ringer solution. Isolated glomeruli were incubated in 36.5 μg/ml Octadecyl rhodamine B chloride (R18, O246; Thermo Fisher Scientific) for 15 minutes, then washed in 4% Ringer BSA to remove unbound R18 followed by 15 minutes’ incubation in 30 μg/ml Alexa Fluor 488-BSA (A13100; Thermo Fisher Scientific). An individual glomerulus was trapped on a custom-made petri dish and the perfusate was switched from 30 μg/ml labeled 488-BSA to 30 μg/ml unlabeled BSA. A Nikon Ti-E inverted confocal microscope (Nikon Instruments Inc., Melville, NY) was used to capture the fluorescence intensity. The rate of decline in fluorescence intensity within the loop of the capillaries for the first minute was used to calculate Ps’alb as previously described.^24^

Please see the Supplementary Methods for details regarding use of electron microscopy, fluorescence-activated cell sorting, RNA extraction, real-time PCR, and TaqMan qPCR array.

### Protein extraction

Isolated glomeruli were homogenized in PBS. Three freeze-thaw cycles were performed to break the cell membranes, and the homogenates were centrifuged for 5 minutes at 5000*g*, at 2 to 8 °C. The supernatant was removed and either assayed immediately or stored at −80 °C.

### Syndecan-4 ELISA

The concentrations of SDC4S7 (ectodomain) in mouse glomerular lysate, plasma, and urine were quantified using a sandwich enzyme immunoassay (mouse SDC4 ELISA kit, CSB-EL020891MO; Cusabio, Houston, TX) according to the manufacturer’s instructions. The concentration of SDC4 was normalized to total protein content as applicable. The fold change of diabetic with MMPI relative to diabetic vehicle was calculated to enable pooling of results from different experiments.

### MMP2 and -9 activity assays

MMP2 and -9 Biotrak Activity AssaysS3 (GE Healthcare Life Sciences, Buckinghamshire, UK) provide precise quantitation of active MMP2 and -9 in plasma, urine, and tissue homogenates. The assays were carried out according to the manufacturer’s instructions. The concentrations of active MMP2 and -9 were normalized to total protein or creatinine concentration as applicable.

### Lectin staining

Paraffin-embedded kidney sections (5 μm) were dewaxed in Histo-Clear (National Diagnostics, Charlotte, NC) followed by rehydration in graded ethanol and a wash in PBS. The sections were incubated in blocking buffer (1% BSA in PBS containing 0.5% Tween) for 30 minutes, followed by endogenous biotin blocking using a streptavidin/biotin blocking kit (SP-2002; Vector Laboratories, Burlingame, CA). After 2 washes, the sections were incubated with biotinylated MOA (2 mg/ml) 1:100, pH 6.8, overnight at 4 °C. Buffer only was used as a negative control. After 4 washes, the sections were incubated with streptavidin AF488 (1:500, S32354; Thermo Fisher Scientific), pH 6.8, for 1 hour at room temperature. The nuclei were counterstained with 4′,6-diamidino-2-phenylindole (Invitrogen, Thermo Fisher Scientific) and the cell membrane labeled with R18 (1:1000, O246; Thermo Fisher Scientific) were incubated for 10 minutes. After a 2-minute wash in PBS, the coverslips were mounted in Vectashield mounting medium (Vector Laboratories) and examined using either an AF600 LX wide-field fluorescence microscope (Leica Microsystems, Milton Keynes, UK) or a Leica SP5-II confocal laser scanning microscope attached to a Leica DMI 6000 inverted epifluorescence microscope.

### Endothelial glycocalyx depth: peak-to-peak analysis

The peak-to-peak assessment was carried out as previously described.^39^^,^^40^ A line (white arrow in Figure 1ei) was drawn from the inside to the outside of the capillary loop crossing the glycocalyx first followed by the R18 endothelial membrane. The line is drawn perpendicular to the endothelial glycocalyx and the cell membrane label to get the maximum consistent depth of the glycocalyx. Fluorescence intensity profiles were then generated for both the SDC4 and MOA components of the endothelial glycocalyx and endothelial cell label. The distance between the peak signals from the SDC4/MOA-488 and the R18 labels (peak-to-peak) is an index of glycocalyx thickness. Peak-to-peak was determined from an average of 3 lines in a loop, 3 loops in a glomerulus, 3 glomeruli in a mouse, and *n* = 5 mice in each group.

### Statistical analysis

Data are expressed as the mean ± SEM. Fold change is defined as the ratio between the 2 groups specified in the text. Normality was assessed using GraphPad Prism 5 (GraphPad Software, La Jolla, CA) Kolmogorov-Smirnov test. Normally distributed data were compared using Student *t* tests for 2 groups and analysis of variance for multiple groups. If 1-way analysis of variance indicated a significant difference, the Bonferroni *post hoc* test was used to assess differences between groups. Where normality could not be demonstrated, the Mann-Whitney test was used for comparing between 2 groups. A *P* value of <0.05 indicated statistical significance.

## Disclosure

All the authors declared no competing interests.

## Supplementary Material

Supplementary Methods and References

Figure S1 Early diabetic kidney disease (DKD) is associated with reduced podocyte glycocalyx but not change in other podocyte or GBM parameters. Control and diabetic mice were perfusion-fixed for electron microscopy with cacodylate buffer containing glutaraldehyde and Alcian blue. (**Ai,ii**) Representative electron micrographs of the glomerular capillary wall are shown at lower and higher magnification. The measurements were carried out on 3 capillary loops per glomerulus and 2 to 3 glomeruli were used per mouse. Labels indicate podocyte glycocalyx (pGLX), (**1**) basement membrane (GBM), (**2**) podocyte slit diaphragm width, and (**3**) podocyte foot process width. Bar =200 nm. Quantification of (**A,Bi**) pGLX depth (control, 23.70 ± 2.341, *n* = 5 mice; diabetes, 15.06 ± 1.713, *n* = 5 mice; **P* = 0.0176) and (**A,Bii**) percentage podocyte with GLX coverage (control, 97.78 ± 2.222, *n* = 5 mice; diabetes, 79.28 ± 10.57, *n* = 5 mice; nonsignificant [NS]); (**A,C**) GBM thickness (control, 134.2 ± 3.047, *n* = 5; diabetes, 138.2 ± 10.89, *n* = 5 mice; NS); (**A,D**) podocyte slit diaphragm width (control, 40.48 ± 1.914, *n* = 5 mice; diabetes, 39.79 ± 3.243, *n* = 5 mice; NS); (**A,E**) podocyte foot process width (control, 251.8 ± 17.07, *n* = 5 mice; diabetes, 264.9 ± 20.16, *n* = 5 mice; NS). (**F,G**) Picrosirius red staining was carried out on control and diabetic kidney sections and representative immunohistochemistry images demonstrate no change in collagen deposition in diabetic glomeruli when compared with control (a minimum of 3 glomeruli were analyzed per mouse; control, 836,500 ± 175,700, *n* = 5 mice; diabetes, 1,060,000 ± 339,200, *n* = 5; NS). Each dot or square on the graph represents a mouse. Data are expressed as mean ± SEM and unpaired Student *t* test at week 9 post-STZ was used for statistical analysis.

Figure S2 Isolation of mouse glomerular endothelial cells. Mouse glomeruli were isolated by sieving and were digested with collagenase. The single cells obtained were immunolabeled with anti-CD31 antibody. The cells were also stained with propidium iodide to exclude the dead ones. Propidium iodide–negative and CD31-positive cells (within the gate) were sorted by a flow cytometry cell sorter to produce pure population of glomerular endothelial cells. The mRNA expression of VEGFR2 (specific for endothelial cells), nephrin (specific for podocyte), and PDGFR2 (specific for mesangial cells), relative to GAPDH was determined. The representative graph shows glomerular endothelial cells had high expression of VEGFR2 but were negative for nephrin and PDGFR2 confirming no or minimal contamination with either podocytes or mesangial cells, *n* = 1 mouse.

Figure S3 Mice were injected i.v. with 0.05 mg/mouse SDC4 antibody (100 μl). After 18 minutes of incubation, cardiac perfusion with Ringer was performed to flush out unbound antibodies. Kidney was snap frozen for immunofluorescence staining. (**A**) Frozen kidney sections were incubated with secondary antibody to SDC4 (Alexa 488, green) and DAPI (blue). Representative images show that the SDC4 antibody localized predominantly to the glomeruli in the SDC4 injected mice and no SDC4 staining can be seen in the PBS-injected mice. (**B**) Frozen kidney sections from SDC4 antibody (red and green) injected mice were immunostained with CD31 (endothelial, green), podocin (podocytes, red), and DAPI (blue). Representative confocal images show SDC4 colocalizes predominantly with endothelial and not podocyte markers.

Figure S4 Blockade of MMP2 and -9 restored diabetes-induced podocyte glycocalyx loss without affecting other podocyte parameters. Diabetic (Dia) ± MMPI or vehicle (Veh)-treated mice were perfusion-fixed for electron microscopy with cacodylate buffer containing glutaraldehyde and Alcian blue. (**Ai,ii**) Representative electron micrographs of the glomerular capillary wall are shown at lower and higher magnification. The measurements were carried out on 3 capillary loops per glomerulus and 2 to 3 glomeruli were used per mouse. Labels indicate podocyte glycocalyx (pGLX), (**1**) basement membrane (GBM) , (**2**) podocyte slit diaphragm width, and (**3**) podocyte foot process width. Bar = 100 nm. Quantification of (**A,Bi**) pGLX depth (Dia Veh, 14.88 ± 1.121, *n* = 5 mice; Dia MMPI, 25.16 ± 2.777, *n* = 6 mice; **P* = 0.0112) and (**A,Bii**) percentage podocyte with GLX coverage (Dia Veh, 90.60 ± 8.402, *n* = 5 mice; Dia MMPI, 97.30 ± 1.746, *n* = 6 mice; nonsignificant [NS]); (**A,C**) GBM thickness (Dia Veh, 154.1 ± 9.837, *n* = 5 mice; Dia MMPI, 149.3 ± 7.904, *n* = 6 mice; NS); (**A,D**) podocyte slit diaphragm width (Dia Veh, 40.12 ± 4.965, *n* = 5 mice; Dia MMPI, 35.42 ± 1.155, *n* = 6 mice; NS); (**A,E**) podocyte foot process width (Dia Veh, 302.5 ± 26.76, n = 5 mice; Dia MMPI, 244.3 ± 13.21, *n* = 6 mice; NS). Each dot or square on the graph represents a mouse. Data are expressed as the mean ± SEM and unpaired Student *t* test at week 9 post-STZ was used for statistical analysis.

Table S1 List of glycocalyx-related genes represented on custom-designed TaqMan qPCR array.

Table S2 Expression Suite software and 2^−ΔΔCT^ method followed by Student *t* test were used to analyze the array data and both methods showed a significant increase in *S**dc**1*, *-3*, and *-4*, and *M**mp**14* mRNA expression among other significantly modulated gene expressions.

Table S3 List of primer probes used in this study.

Table S4 Absolute values of albumin, creatinine, and albumin creatinine ratio from 3 independent experiments carried out on diabetic (Dia) + MMPI or vehicle (Veh) mice.
