# Supplementary material for: Blocking matrix metalloproteinase-mediated syndecan-4 shedding restores the endothelial glycocalyx and glomerular filtration barrier function in early diabetic kidney disease
Source: Kidney Int. 2020 May;97(5):951–65. doi: 10.1016/j.kint.2019.09.035 (PMC7184681; doi:10.1016/j.kint.2019.09.035)
Supplement: Supplementary Methods and References [file mmc1.docx]

**Supplementary Methods**

**Tissue collection in Type 1 diabetes model**

Control, diabetic with or without the MMPI inhibitor/vehicle were culled for tissue collection at 8 or 9 weeks post STZ injection. MMP inhibitor is an N-sulfonyl amino acid derivative modified in the amino acid residue and the sulfonamide moiety. Studies carried out on its enzymatic activity have shown that the sulfonamide derivatives containing biaryl, triple bond, tetrazole, or amide were selective inhibitors of type IV collagenase (MMP-9 and MMP-2) in vitro as well as in vivo.^S1^ This highly potent and selective inhibitor blocks MMP2 (IC50 = 310 nM) and 9 (IC50 = 240 nM) without affecting other metalloproteinases. ^S1^ MMPI is easily absorbed across the gastrointestinal tract. ^S2, S3^ It has been shown to provide protective effects in several disease states: reduce brain edema and preserves the Blood Brain Barrier in a brain injury model, ^S4^ ameliorates pathogenesis and improves skeletal muscle regeneration in muscular dystrophy ^S2^ and attenuates albuminuria in an aldosterone-induced kidney disease model. ^S3^

For tissue collection, some mice were anaesthetised with 3.5% isoflurane in 1L/min O_2_. A midline laparotomy was performed, and the abdominal aorta was cannulated with PE-10 tubing (Becton Dickinson, 427400, Franklin Lakes, NJ) to flush both kidneys with Ringer solution (NaCl, 132mM; KCl, 4.6mM; MgSO4-7H2O 1.27mM; CaCl2-2H2O 2mM; NaHCO3, 25mM; D(+)glucose, 5.5mM; N-2-hydroxyethylpiperazine-N’-2-ethanesulphonic (HEPES) acid, 3.07mM; HEPES sodium salt, 1.9mM, pH 7.40). One kidney was removed for glomerular sieving as previously ^S5^ and the other kidney was perfusion-fixed with a solution containing 2.5% glutaraldehyde, 0.1M cacodylate and 1% Alcian blue for transmission electron microscopy to quantifying endothelial glycocalyx depth and coverage. ^S6^ In other mice, instead of perfusion, blood was collected for plasma isolation and the kidneys used for glomerular sieving. Some mice were intravenously (iv) injected with 0.05 mg/mouse SDC4 antibody. After 18 min of incubation, cardiac perfusion with Ringer was performed to flush out unbound antibody. Kidney was snap frozen for immunofluorescence staining.

**Electron microscopy**

Electron micrographs were taken using a Technai 12 electron microscope (FEI, Hillsboro, Oregon) and image analysis was carried out as previously in 3 capillary loops per glomerulus and 2–3 glomeruli per animal. ^S5, S6^ Briefly, ImageJ software (SciJava software ecosystem, Maryland, USA) was used to overlay a grid onto the electron micrograph. The anatomical distance from the luminal phospholipid at sequential grid intersections to the furthest point of glycocalyx was measured as glycocalyx depth. A glycocalyx depth ≤ 10nm was considered uncovered and this was expressed as a percentage of total measurements taken on a grid section. Glycocalyx coverage expressed as a percentage = 100% - glycocalyx uncovered%. GBM thickness, podocyte foot process width and slit diaphragm width were also measured. Observers were blinded to sample identity before analysis.

**Fluorescence Activated Cell Sorting (FACS)**

Mouse glomeruli were collected from 75µm pore sieves as previously. ^S5^ The glomeruli were digested with 1mg/ml collagenases (1, V, VI) (Sigma-Aldrich, Dorset, UK) for 1 hour at 37°C in a hybridization oven with constant rotation. After the enzymatic digestion was complete, the digest was passed through a 35µm mesh cell strainer (BD falcon 352235, ThermoFisher Scientific) to give a single cell suspension. After 3 washes, the cell pellet was resuspended in HBSS containing 1mM EDTA, 0.1ul/ml DNAse and 0.3% BSA (Sigma-Aldrich) and immunostained with PE Rat Anti-Mouse CD31 antibody (BD Pharmingen, San Jose, CA) at a 1:50 dilution for 1 hour at 4°C. After 3 washes, the cells were ready for FACS. CD31 positive cells were isolated using a Becton Dickinson Influx Cell Sorter (BD Biosciences, San Jose, CA). In brief, single viable cells were detected based on laser scatter, trigger pulse width and exclusion of the vital dye Propidium Iodide (PI). Cells were excited with 488nm and 552nm lasers and fluorescence emissions were captured for PE (552nm 582/29nm BP) and PI (552nm 710/50 BP) respectively. Controls included were unstained cells and isotype control (Rat IgG2a, κ Isotype Control, FITC Goat Anti-Rat Igs, BD Pharmingen).

**RNA extraction**

RNA was extracted from isolated glomeruli using an RNeasy Mini kit (Qiagen, Manchester, UK) according to manufacturer’s instructions. Then 2μg of total RNA was converted to cDNA by a high-capacity RNA to cDNA conversion kit (Applied Biosystems, Foster City, CA, USA), according to the manufacturer's instructions.

**Real-time PCR**

The mRNA expression of relevant genes was quantified according to the manufacturer’s instructions for real-time PCR (StepOnePlus Real-Time PCR System; Applied Biosystems) using TaqMan primer probes (Life Technologies, ThermoFisher Scientific) detailed in supplementary Table 3. PCRs were performed in duplicate. The 2^−ΔΔ^*^CT^* method was used to calculate the fold change, normalized to GAPDH.

**TaqMan qPCR array**

The custom-designed TaqMan Low Density Array (TLDA, Applied Biosystems) is a 384-well microfluidic card that performs 384 simultaneous real-time PCR. We designed an array which included 53 glycocalyx-related and control genes (Table 2, supplementary material). cDNA (1:5 dilution) and TaqMan gene expression master mix (Applied Biosystems) were used to perform the reaction. In brief, the wells of the TaqMan array contained TaqMan gene expression assays that detected the real-time amplification of the specified targets. Relative levels of gene expression were determined from the fluorescence data generated during PCR using a ViiA7 Real-Time PCR System (Applied Biosystems). Expression Suite software (ThermoFisher scientific) which utilises the comparative Cτ (ΔΔCτ) method was used to rapidly and accurately quantify relative gene expression across a large number of genes and samples. For the selected genes of interest, the 2^−ΔΔ^*^CT^* method was also used to calculate fold changes, normalized to GAPDH. Independent *t*-tests with p < 0.05 were used as a screening test, and genes that were significantly modulated (and MMP9 in view of previous results) ^S7^ were selected for further analysis by independent real-time PCR.

**Immunofluorescence staining**

5 µm cut frozen kidney sections were fixed with 4% paraformaldehyde. The sections were incubated in blocking solution [1% bovine serum albumin (BSA) in PBS containing 0.1% Tween] for 1 hour. Some sections from the SDC4 (Purified Rat Anti-Mouse Syndecan-4, Clone KY/8.2, BD Biosciences) injected mice were incubated with Alexa Fluor™ 488 secondary antibody (Molecular Probes; Life Technologies). Others were incubated with primary antibodies to either CD31 (FITC Rat Anti-Mouse CD31MEC13.3, BD Biosciences at 1:100 dilution) or podocin (P0372, Sigma-Aldrich, at 1:500 dilution) in blocking buffer for 40 min at room temperature. Primary antibody binding was detected by using Alexa Fluor™ 488 and 633 secondary antibodies (Molecular Probes; Life Technologies). After 3 washes, the nuclei were counterstained with 4′,6-diamidino-2-phenylindole dihydrochloride (DAPI; Invitrogen; Life Technologies). For peak to peak analysis, the sections were incubated with an endothelial cell membrane label R18 (O246, ThermoFisher Scientific, at 1:1000 dilution) for 10 min. After a 2 min wash in PBS, the coverslips were mounted in Vectashield mounting medium (Vector Laboratories, Peterborough, UK) and examined using either an AF600 LX wide-field fluorescence microscope (Leica Microsystems, Milton Keynes, UK) or a Leica SP5-II confocal laser scanning microscope attached to a Leica DMI 6000 (Leica Microsystems) inverted epifluorescence microscope.

**Picrosirius Red Staining**

Picrosirius Red Staining was carried out as. ^S8^ Mouse paraffin tissue sections were hydrated followed by counterstaining in a 0.1% (w/v) Direct Red 80 powder (SigmaAldrich) in a 1.3% saturated aqueous solution of picric acid (VWR Chemicals) for 90 min at room temperature, then dehydrated and mounted. Images were taken using a bright-field microscope. Images were quantified using Image J. A minimum of 3 glomeruli were analysed per mouse and n=5 mice per group. Images were converted to RGB images and colour deconvolution was performed. Red staining channel was then converted to 8-bit gray scale image and colour inverted. Total of 4 background mean gray value measurements were taken. Tracing tool was used to draw around glomerulus to measure area and integrated density. Corrected Total Cell Fluorescence (CTCF) was then calculated by subtracting the average of background mean gray values multiplied by area of glomerulus from glomerular integrated density. CTCF values for each mouse were then averaged and plotted.

**Supplementary references**

S1. Tamura Y, Watanabe F, Nakatani T*, et al.* Highly selective and orally active inhibitors of type IV collagenase (MMP-9 and MMP-2): N-sulfonylamino acid derivatives. *J Med Chem* 1998; **41:** 640-649.

S2. Li H, Mittal A, Makonchuk DY*, et al.* Matrix metalloproteinase-9 inhibition ameliorates pathogenesis and improves skeletal muscle regeneration in muscular dystrophy. *Human molecular genetics* 2009; **18:** 2584-2598.

S3. Butler MJ, Ramnath R, Kadoya H*, et al.* Aldosterone induces albuminuria via matrix metalloproteinase-dependent damage of the endothelial glycocalyx. *Kidney international* 2019; **95:** 94-107.

S4. Yamaguchi M, Jadhav V, Obenaus A*, et al.* Matrix metalloproteinase inhibition attenuates brain edema in an in vivo model of surgically-induced brain injury. *Neurosurgery* 2007; **61:** 1067-1075; discussion 1075-1066.

S5. Desideri S, Onions KL, Qiu Y*, et al.* A novel assay provides sensitive measurement of physiologically relevant changes in albumin permeability in isolated human and rodent glomeruli. *Kidney international* 2018.

S6. Oltean S, Qiu Y, Ferguson JK*, et al.* Vascular Endothelial Growth Factor-A165b Is Protective and Restores Endothelial Glycocalyx in Diabetic Nephropathy. *J Am Soc Nephrol* 2015; **26:** 1889-1904.

S7. Ramnath R, Foster RR, Qiu Y*, et al.* Matrix metalloproteinase 9-mediated shedding of syndecan 4 in response to tumor necrosis factor alpha: a contributor to endothelial cell glycocalyx dysfunction. *FASEB J* 2014; **28:** 4686-4699.

S8. Onions KL, Gamez M, Buckner NR*, et al.* VEGFC Reduces Glomerular Albumin Permeability and Protects Against Alterations in VEGF Receptor Expression in Diabetic Nephropathy. *Diabetes* 2019; **68:** 172-187.
