# Supplementary material for: Blocking matrix metalloproteinase-mediated syndecan-4 shedding restores the endothelial glycocalyx and glomerular filtration barrier function in early diabetic kidney disease
Source: Kidney Int. 2020 May;97(5):951–65. doi: 10.1016/j.kint.2019.09.035 (PMC7184681; doi:10.1016/j.kint.2019.09.035)
Supplement: Figure S1 — Early diabetic kidney disease (DKD) is associated with reduced podocyte glycocalyx but not change in other podocyte or GBM parameters. Control and diabetic mice were perfusion-fixed for electron microscopy with cacodylate buffer containing glutaraldehyde and Alcian blue. (Ai,ii) Representative electron micrographs of the glomerular capillary wall are shown at lower and higher magnification. The measurements were carried out on 3 capillary loops per glomerulus and 2 to 3 glomeruli were used per mouse. Labels indicate podocyte glycocalyx (pGLX), (1) basement membrane (GBM), (2) podocyte slit diaphragm width, and (3) podocyte foot process width. Bar =200 nm. Quantification of (A,Bi) pGLX depth (control, 23.70 ± 2.341, n = 5 mice; diabetes, 15.06 ± 1.713, n = 5 mice; *P = 0.0176) and (A,Bii) percentage podocyte with GLX coverage (control, 97.78 ± 2.222, n = 5 mice; diabetes, 79.28 ± 10.57, n = 5 mice; nonsignificant [NS]); (A,C) GBM thickness (control, 134.2 ± 3.047, n = 5; diabetes, 138.2 ± 10.89, n = 5 mice; NS); (A,D) podocyte slit diaphragm width (control, 40.48 ± 1.914, n = 5 mice; diabetes, 39.79 ± 3.243, n = 5 mice; NS); (A,E) podocyte foot process width (control, 251.8 ± 17.07, n = 5 mice; diabetes, 264.9 ± 20.16, n = 5 mice; NS). (F,G) Picrosirius red staining was carried out on control and diabetic kidney sections and representative immunohistochemistry images demonstrate no change in collagen deposition in diabetic glomeruli when compared with control (a minimum of 3 glomeruli were analyzed per mouse; control, 836,500 ± 175,700, n = 5 mice; diabetes, 1,060,000 ± 339,200, n = 5; NS). Each dot or square on the graph represents a mouse. Data are expressed as mean ± SEM and unpaired Student t test at week 9 post-STZ was used for statistical analysis. [file mmc2.pdf]

Figure S1

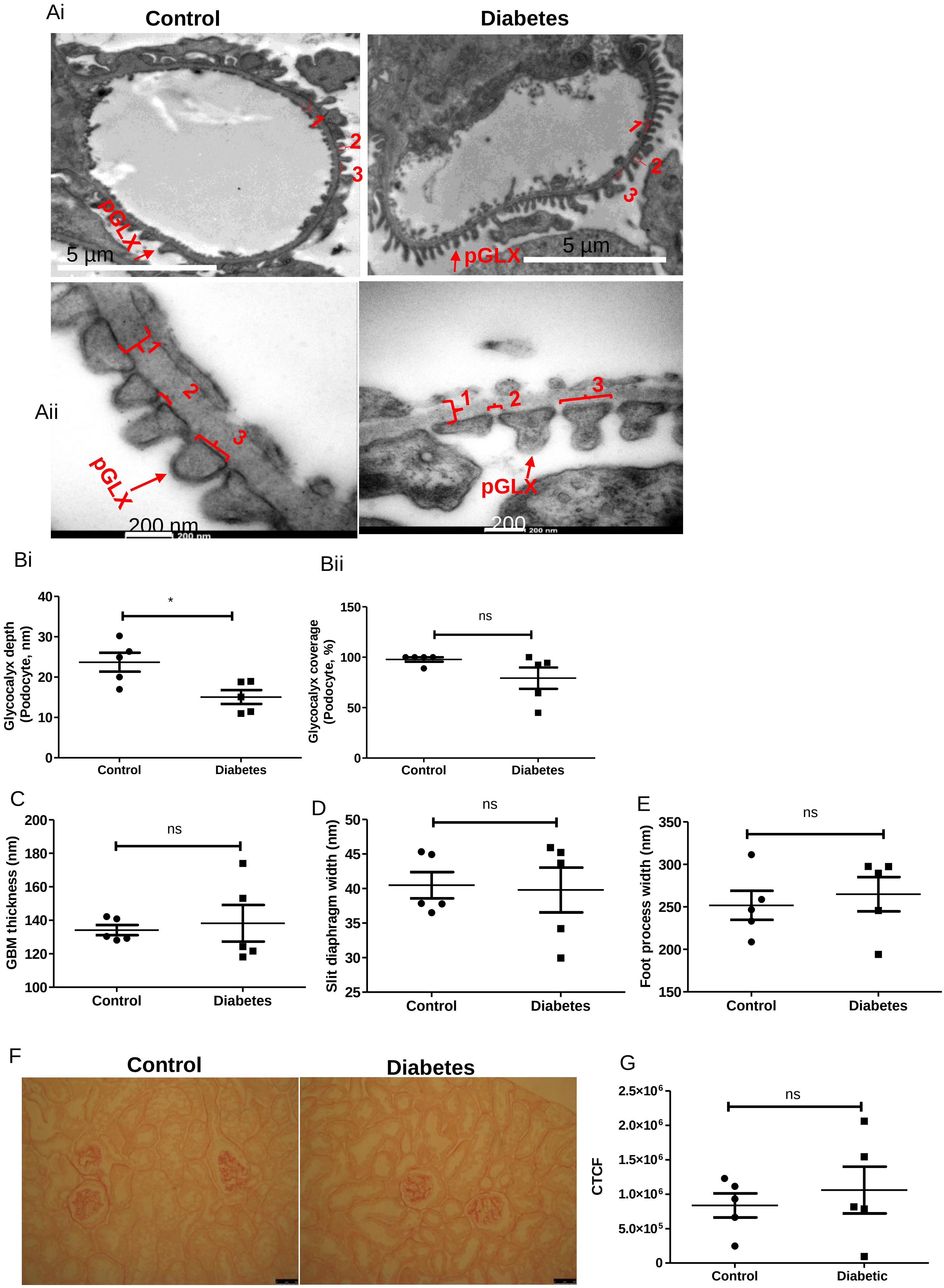

Ai
Control
Diabetes
pGLX
5 µm
1
2
3
Aii
200 nm
Bi
Glycocalyx depth (Podocyte, nm)
*
Control
Diabetes
Bii
Glycocalyx coverage (Podocyte, %)
ns
C
GBM thickness (nm)
D
Slit diaphragm width (nm)
E
Foot process width (nm)
F
G
CTCF
Diabetic

Supplementary Figure 1. Early diabetic kidney disease (DKD) is associated with reduced podocyte glycocalyx but not change in other podocyte or GBM parameters. Control and diabetic mice were perfusion-fixed for electron microscopy with cacodylate buffer containing glutaraldehyde and Alcian blue. Representative electron micrographs of the glomerular capillary wall are shown at lower and higher magnification (Ai, ii). The measurements were carried out on 3 capillary loops per glomerulus and 2–3 glomeruli were used per mouse. Labels indicate podocyte glycocalyx (pGLX), basement membrane (GBM, 1), podocyte slit diaphragm width (2) and podocyte foot process width (3) (scale bar =200nm). Quantification of (A, Bi) pGLX depth (control 23.70 ± 2.341 n=5 mice, diabetes 15.06 ± 1.713 n=5 mice, *p=0.0176) and (A, Bii) percentage podocyte with GLX coverage (control 97.78 ± 2.222 n=5 mice, diabetes 79.28 ± 10.57 n=5 mice, non significant (ns)); (A, C) GBM thickness (control 134.2 ± 3.047 n=5, diabetes 138.2 ± 10.89 n=5 mice, ns). (A, D) podocyte slit diaphragm width (control 40.48 ± 1.914 n=5 mice, diabetes 39.79 ± 3.243 n=5 mice, ns); (A. E) podocyte foot process width (control 251.8 ± 17.07 n=5 mice, diabetes 264.9 ± 20.16 n=5 mice, ns). (F, G) Picro Sirius red staining was carried out on control and diabetic kidney sections and representative immunohistochemistry images demonstrate no change in collagen deposition in diabetic glomeruli when compared to control (a minimum of 3 glomeruli were analysed per mouse, control 836500 ± 175700 n=5 mice, diabetes 1060000 ± 339200 n=5(ns)). Each dot or square on the graph represents a mouse. Data is expressed as the mean ± SEM and unpaired *t* test at week 9 post STZ was used for statistical analysis.
