# Supplementary figures and images for: Blocking matrix metalloproteinase-mediated syndecan-4 shedding restores the endothelial glycocalyx and glomerular filtration barrier function in early diabetic kidney disease
Source: Kidney Int. 2020 May;97(5):951–65. doi: 10.1016/j.kint.2019.09.035 (PMC7184681; doi:10.1016/j.kint.2019.09.035)

Figure S2

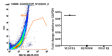

Supplement: Figure S2 — Isolation of mouse glomerular endothelial cells. Mouse glomeruli were isolated by sieving and were digested with collagenase. The single cells obtained were immunolabeled with anti-CD31 antibody. The cells were also stained with propidium iodide to exclude the dead ones. Propidium iodide–negative and CD31-positive cells (within the gate) were sorted by a flow cytometry cell sorter to produce pure population of glomerular endothelial cells. The mRNA expression of VEGFR2 (specific for endothelial cells), nephrin (specific for podocyte), and PDGFR2 (specific for mesangial cells), relative to GAPDH was determined. The representative graph shows glomerular endothelial cells had high expression of VEGFR2 but were negative for nephrin and PDGFR2 confirming no or minimal contamination with either podocytes or mesangial cells, n = 1 mouse. [file mmc3.pdf]
