# Supplementary material for: Blocking matrix metalloproteinase-mediated syndecan-4 shedding restores the endothelial glycocalyx and glomerular filtration barrier function in early diabetic kidney disease
Source: Kidney Int. 2020 May;97(5):951–65. doi: 10.1016/j.kint.2019.09.035 (PMC7184681; doi:10.1016/j.kint.2019.09.035)
Supplement: Figure S3 — Mice were injected i.v. with 0.05 mg/mouse SDC4 antibody (100 μl). After 18 minutes of incubation, cardiac perfusion with Ringer was performed to flush out unbound antibodies. Kidney was snap frozen for immunofluorescence staining. (A) Frozen kidney sections were incubated with secondary antibody to SDC4 (Alexa 488, green) and DAPI (blue). Representative images show that the SDC4 antibody localized predominantly to the glomeruli in the SDC4 injected mice and no SDC4 staining can be seen in the PBS-injected mice. (B) Frozen kidney sections from SDC4 antibody (red and green) injected mice were immunostained with CD31 (endothelial, green), podocin (podocytes, red), and DAPI (blue). Representative confocal images show SDC4 colocalizes predominantly with endothelial and not podocyte markers. [file mmc4.pptx]

## Slide 1
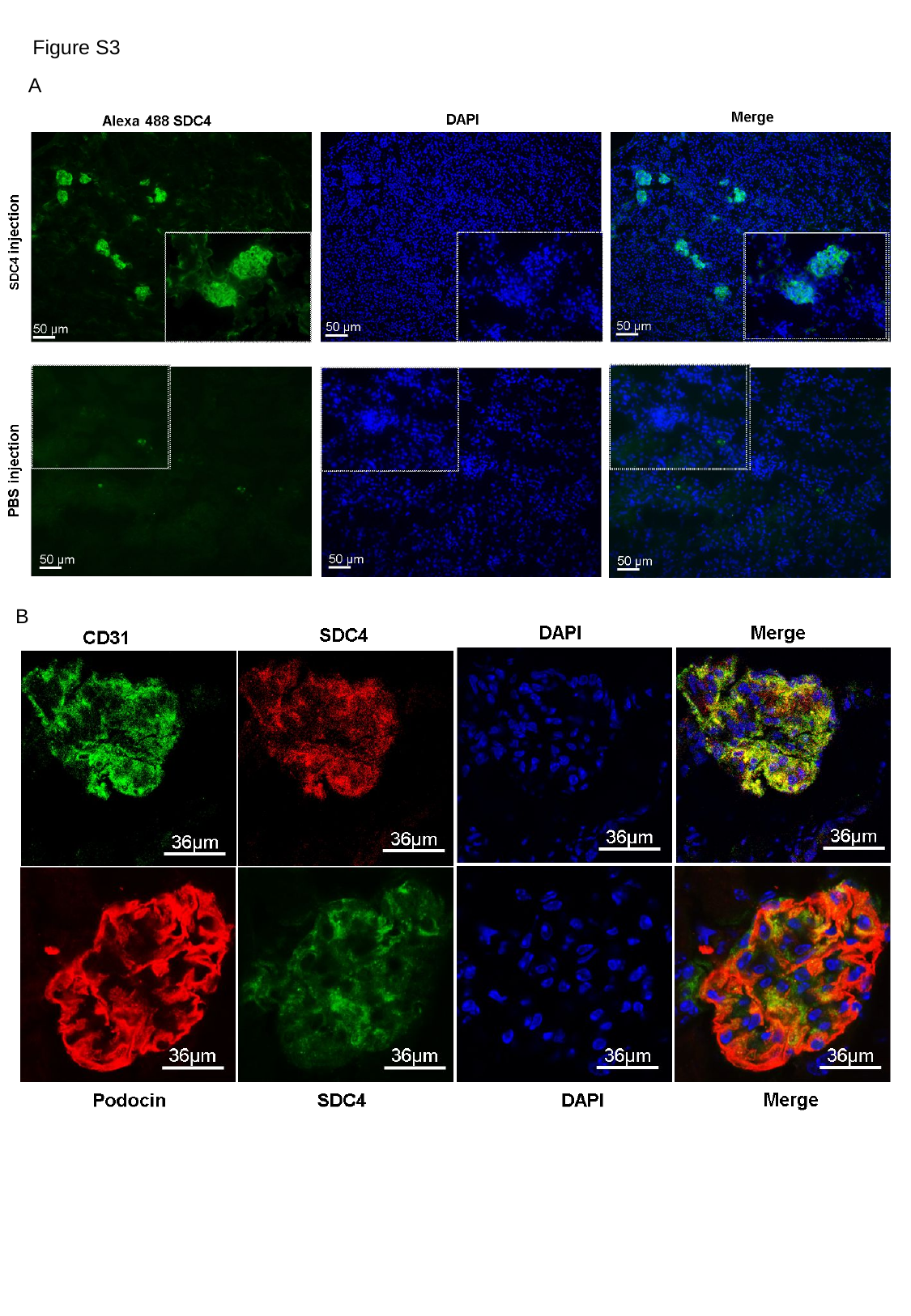

Figure S3
A
B

## Slide 2
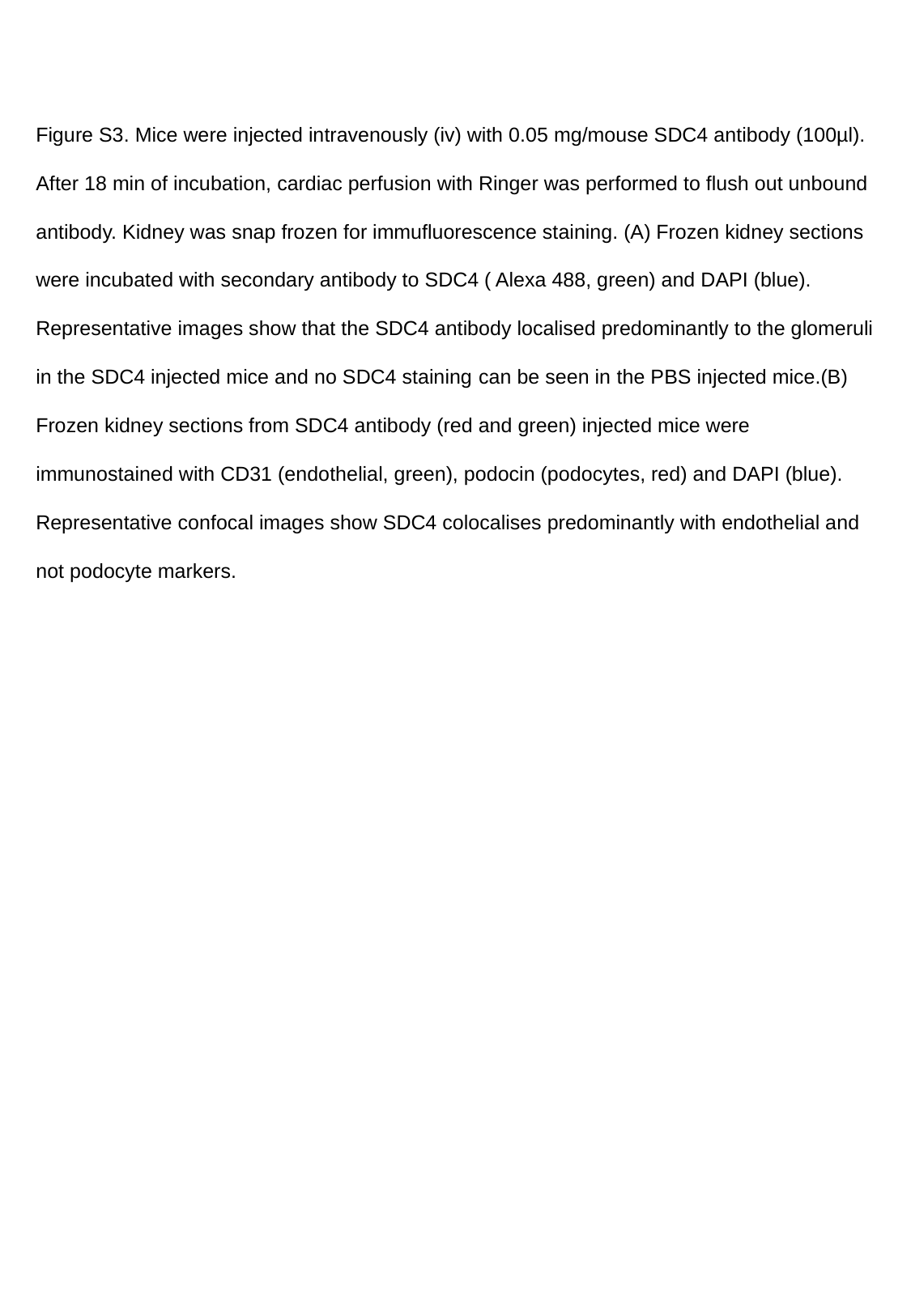

Figure S3. Mice were injected intravenously (iv) with 0.05 mg/mouse SDC4 antibody (100µl). After 18 min of incubation, cardiac perfusion with Ringer was performed to flush out unbound antibody. Kidney was snap frozen for immufluorescence staining. (A) Frozen kidney sections were incubated with secondary antibody to SDC4 ( Alexa 488, green) and DAPI (blue). Representative images show that the SDC4 antibody localised predominantly to the glomeruli in the SDC4 injected mice and no SDC4 staining can be seen in the PBS injected mice.(B) Frozen kidney sections from SDC4 antibody (red and green) injected mice were immunostained with CD31 (endothelial, green), podocin (podocytes, red) and DAPI (blue). Representative confocal images show SDC4 colocalises predominantly with endothelial and not podocyte markers.
