# Supplementary material for: Blocking matrix metalloproteinase-mediated syndecan-4 shedding restores the endothelial glycocalyx and glomerular filtration barrier function in early diabetic kidney disease
Source: Kidney Int. 2020 May;97(5):951–65. doi: 10.1016/j.kint.2019.09.035 (PMC7184681; doi:10.1016/j.kint.2019.09.035)
Supplement: Figure S4 — Blockade of MMP2 and -9 restored diabetes-induced podocyte glycocalyx loss without affecting other podocyte parameters. Diabetic (Dia) ± MMPI or vehicle (Veh)-treated mice were perfusion-fixed for electron microscopy with cacodylate buffer containing glutaraldehyde and Alcian blue. (Ai,ii) Representative electron micrographs of the glomerular capillary wall are shown at lower and higher magnification. The measurements were carried out on 3 capillary loops per glomerulus and 2 to 3 glomeruli were used per mouse. Labels indicate podocyte glycocalyx (pGLX), (1) basement membrane (GBM) , (2) podocyte slit diaphragm width, and (3) podocyte foot process width. Bar = 100 nm. Quantification of (A,Bi) pGLX depth (Dia Veh, 14.88 ± 1.121, n = 5 mice; Dia MMPI, 25.16 ± 2.777, n = 6 mice; *P = 0.0112) and (A,Bii) percentage podocyte with GLX coverage (Dia Veh, 90.60 ± 8.402, n = 5 mice; Dia MMPI, 97.30 ± 1.746, n = 6 mice; nonsignificant [NS]); (A,C) GBM thickness (Dia Veh, 154.1 ± 9.837, n = 5 mice; Dia MMPI, 149.3 ± 7.904, n = 6 mice; NS); (A,D) podocyte slit diaphragm width (Dia Veh, 40.12 ± 4.965, n = 5 mice; Dia MMPI, 35.42 ± 1.155, n = 6 mice; NS); (A,E) podocyte foot process width (Dia Veh, 302.5 ± 26.76, n = 5 mice; Dia MMPI, 244.3 ± 13.21, n = 6 mice; NS). Each dot or square on the graph represents a mouse. Data are expressed as the mean ± SEM and unpaired Student t test at week 9 post-STZ was used for statistical analysis. [file mmc5.pdf]

Figure S4

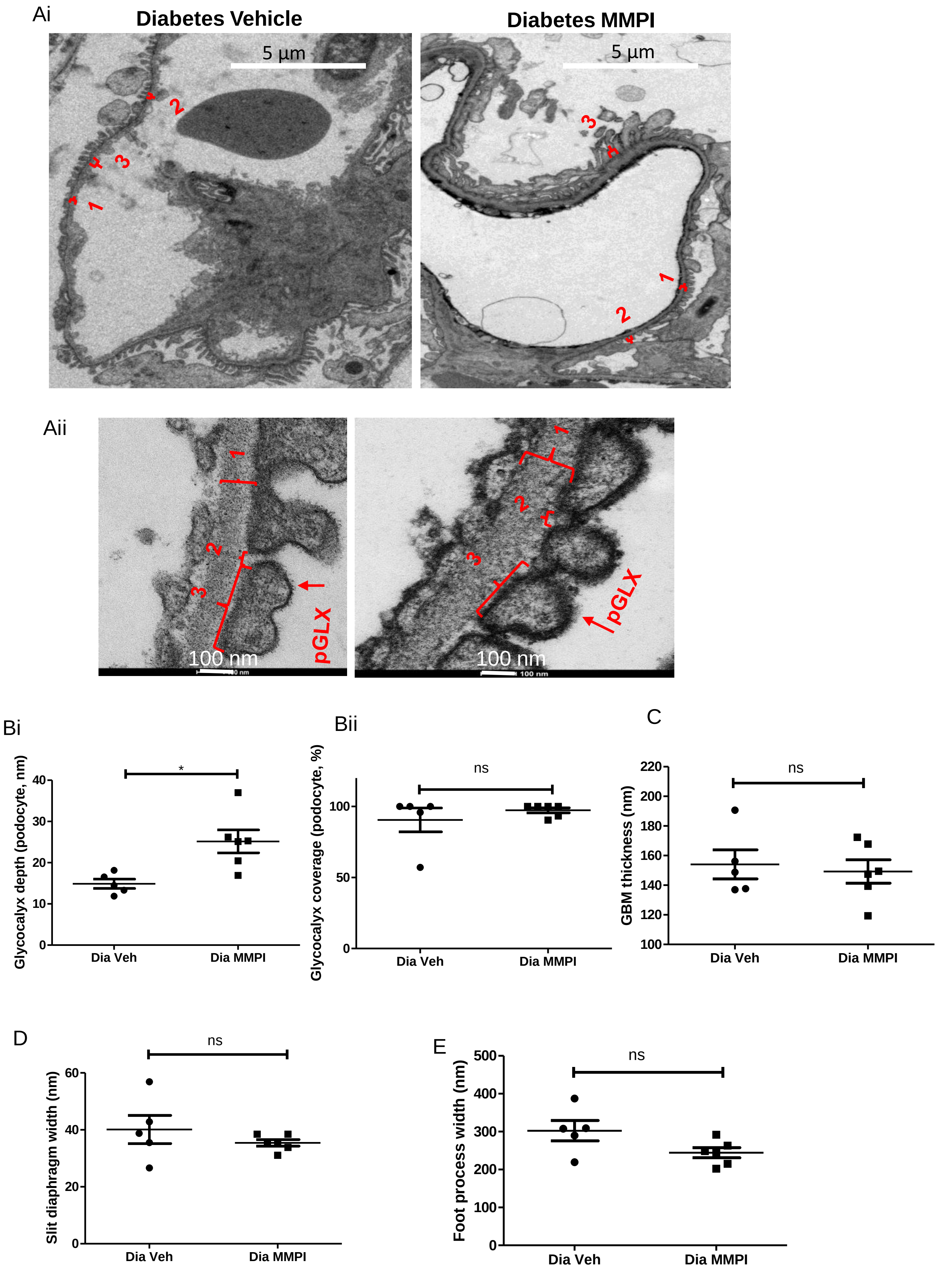
Ai
Diabetes Vehicle
Diabetes MMPI
5 µm
5 µm
1
2
3
Aii
1
2
3
pGLX
100 nm
100 nm
Bi
Glycocalyx depth (podocyte, nm)
*
0
10
20
30
40
Dia Veh
Dia MMPI
Bii
Glycocalyx coverage (podocyte, %)
ns
0
50
100
Dia Veh
Dia MMPI
C
GBM thickness (nm)
ns
100
120
140
160
180
200
220
Dia Veh
Dia MMPI
D
Slit diaphragm width (nm)
ns
0
20
40
60
Dia Veh
Dia MMPI
E
Foot process width (nm)
ns
0
100
200
300
400
500
Dia Veh
Dia MMPI

Figure S4. Blockade of MMP2 and 9 restored diabetes-induced podocyte glycocalyx loss without affecting other podocyte parameters. Diabetic (Dia) ± MMPI or vehicle (Veh)-treated mice were perfusion-fixed for electron microscopy with cacodylate buffer containing glutaraldehyde and Alcian blue. Representative electron micrographs of the glomerular capillary wall are shown at lower and higher magnification (Ai, ii). The measurements were carried out on 3 capillary loops per glomerulus and 2–3 glomeruli were used per mouse. Labels indicate podocyte glycocalyx (pGLX), basement membrane (GBM, 1), podocyte slit diaphragm width (2) and podocyte foot process width (3) (scale bar =100nm). Quantification of (A, Bi) pGLX depth (Dia Veh 14.88 ± 1.121 n=5 mice, Dia MMPI 25.16 ± 2.777 n=6 mice, *p=0.0112) and (A, Bii) percentage podocyte with GLX coverage (Dia Veh 90.60 ± 8.402 n=5 mice, Dia MMPI 97.30 ± 1.746 n=6 mice, non significant (ns)); (A, C) GBM thickness (Dia Veh 154.1 ± 9.837 n=5 mice, Dia MMPI 149.3 ± 7.904 n=6 mice, ns); (A, D) podocyte slit diaphragm width (Dia Veh 40.12 ± 4.965 n=5 mice, Dia MMPI 35.42 ± 1.155 n=6 mice, ns); (A. E) podocyte foot process width (Dia Veh 302.5 ± 26.76 n=5 mice, Dia MMPI 244.3 ± 13.21 n=6 mice, ns). Each dot or square on the graph represents a mouse. Data is expressed as the mean ± SEM and unpaired *t* test at week 9 post STZ was used for statistical analysis.
