# Supplementary material for: Blocking matrix metalloproteinase-mediated syndecan-4 shedding restores the endothelial glycocalyx and glomerular filtration barrier function in early diabetic kidney disease
Source: Kidney Int. 2020 May;97(5):951–65. doi: 10.1016/j.kint.2019.09.035 (PMC7184681; doi:10.1016/j.kint.2019.09.035)
Supplement: Table S1 — List of glycocalyx-related genes represented on custom-designed TaqMan qPCR array. [file mmc6.pdf]

Table S1

Class	Name	Symbol
Proteoglycans	Syndecan	SDC 1, 2, 3, 4
	Glypican	GPC 1, 5
	Biglycan	BGN
	Heparan sulfate proteoglycan 2	HSPG2
	Versican	VCAN
	Endothelial cell-specific molecule 1	ESM1
	Decorin	DCN
	Lumican	LUM
Proteoglycan degrading enzymes	Matrix metalloproteinase	MMP 2, 3, 7, 9, 14, 16
	ADAM Metallopeptidase Domain	ADAM 17
	ADAM Metallopeptidase With Thrombospondin Type 1 Motif	ADAMTS 1, 4
HS-synthesizing enzymes	Exostoses	EXT 1, 2
	N-deacetylase/N-sulfotransferase	NDST 1, 2
	Heparan sulfate 2-O-sulfotransferase	HS2ST1
	Heparan sulfate 6-O-sulfotransferase	HS6ST 1, 2
HS-degrading/modifying enzymes	Heparanase	HPSE
	Sulfatase	SULF 1, 2
Chondroitin sulfate-synthesizing enzymes	Chondroitin sulfate N-acetylgalactosaminyltransferase	CSGALNACT 1, 2
	Chondroitin sulfate synthase	CHSY
GAG biosynthesis	Carbohydrate Sulfotransferase	CHST 2, 11
Hyaluronan-synthesizing enzymes	Hyaluronan synthase	HAS 1, 2, 3
Hyaluronan-degrading enzymes	Hyaluronoglucosaminidase 1, 2	HYAL 1, 2
Hyaluronan receptors	Hyaluronan Mediated Motility Receptor	HMMR
	CD44 Molecule (Indian Blood Group)	CD44
Sialic acid-modifying enzymes	Glucosamine (UDP-N-acetyl)-2-epimerase	GNE
	N-Acetylneuraminic acid synthase	NANS
	Sialidase	NEU
Sialoglycoproteins	Endomucin	EMCN
	Podocalyxin-like	PODXL 1, 2
Others	Thrombomodulin	THBD
Housekeeping genes	Glyceraldehyde-3-Phosphate Dehydrogenase	GAPDH
	Eukaryotic 18S rRNA	18S
	Actin, β	ACTB

Supplementary Table 1: List of glycocalyx related genes represented on custom-designed TaqMan qPCR array
