# Supplementary material for: Blocking matrix metalloproteinase-mediated syndecan-4 shedding restores the endothelial glycocalyx and glomerular filtration barrier function in early diabetic kidney disease
Source: Kidney Int. 2020 May;97(5):951–65. doi: 10.1016/j.kint.2019.09.035 (PMC7184681; doi:10.1016/j.kint.2019.09.035)
Supplement: Table S2 — Expression Suite software and 2−ΔΔCT method followed by Student t test were used to analyze the array data and both methods showed a significant increase in Sdc1, -3, and -4, and Mmp14 mRNA expression among other significantly modulated gene expressions. [file mmc7.pptx]

## Slide 1
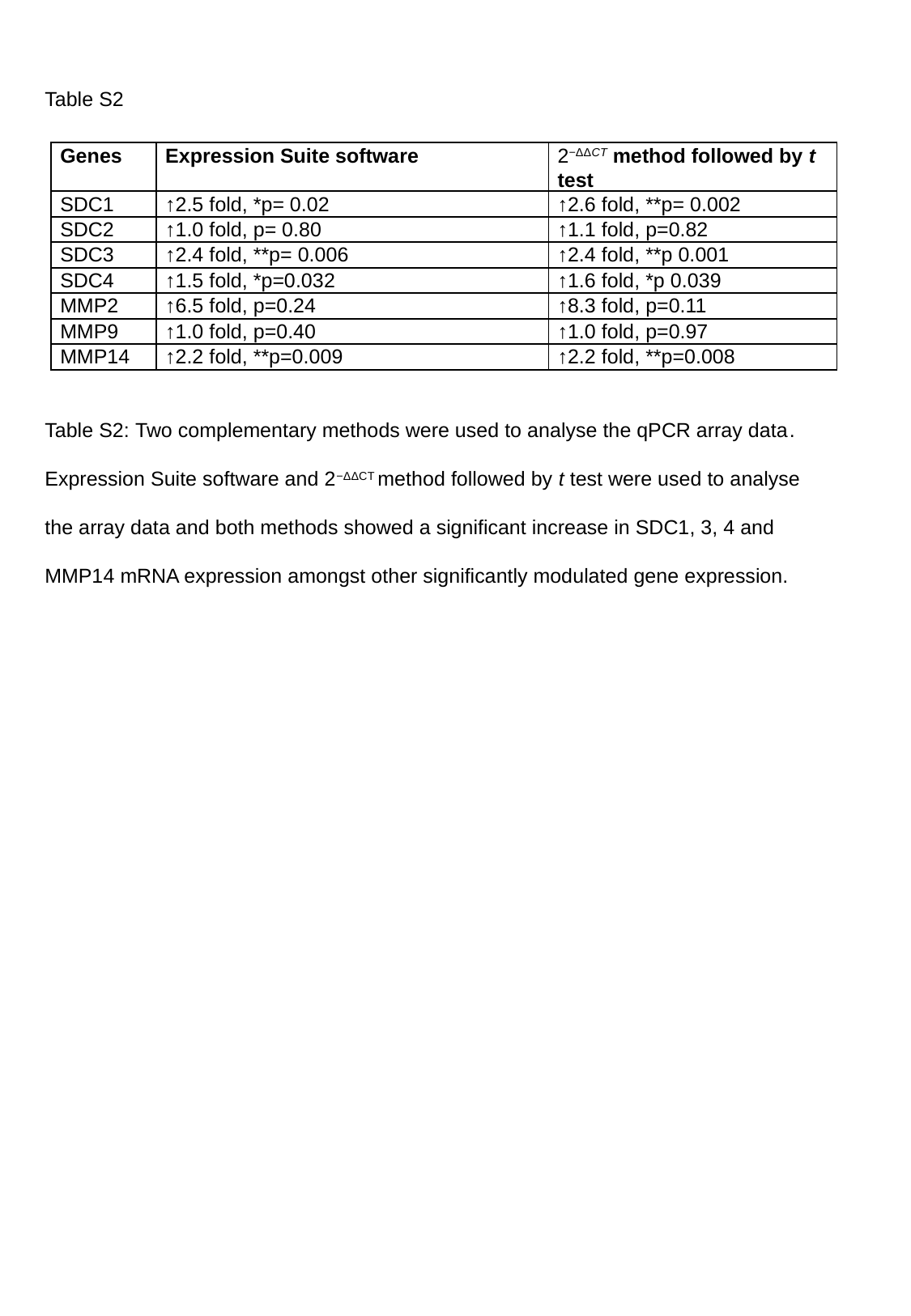

Table S2
| Genes | Expression Suite software | 2−ΔΔCT method followed by t test |
| --- | --- | --- |
| SDC1 | ↑2.5 fold, \*p= 0.02 | ↑2.6 fold, \*\*p= 0.002 |
| SDC2 | ↑1.0 fold, p= 0.80 | ↑1.1 fold, p=0.82 |
| SDC3 | ↑2.4 fold, \*\*p= 0.006 | ↑2.4 fold, \*\*p 0.001 |
| SDC4 | ↑1.5 fold, \*p=0.032 | ↑1.6 fold, \*p 0.039 |
| MMP2 | ↑6.5 fold, p=0.24 | ↑8.3 fold, p=0.11 |
| MMP9 | ↑1.0 fold, p=0.40 | ↑1.0 fold, p=0.97 |
| MMP14 | ↑2.2 fold, \*\*p=0.009 | ↑2.2 fold, \*\*p=0.008 |
Table S2: Two complementary methods were used to analyse the qPCR array data. Expression Suite software and 2−ΔΔCT method followed by t test were used to analyse the array data and both methods showed a significant increase in SDC1, 3, 4 and MMP14 mRNA expression amongst other significantly modulated gene expression.
