# Supplementary material for: Blocking matrix metalloproteinase-mediated syndecan-4 shedding restores the endothelial glycocalyx and glomerular filtration barrier function in early diabetic kidney disease
Source: Kidney Int. 2020 May;97(5):951–65. doi: 10.1016/j.kint.2019.09.035 (PMC7184681; doi:10.1016/j.kint.2019.09.035)
Supplement: Table S3 — List of primer probes used in this study. [file mmc8.pdf]

Table S3

Genes	Interrogated Sequence		Translated Protein	Exon Boundary	Assay Location	Amplicon Length
SDC1	RefSeq	NM_011519.2	NP_035649.1	2--3	387	131
SDC2	RefSeq	NM_008304.2	NP_032330.1	3--4	833	113
SDC3	RefSeq	NM_011520.3	NP_035650.2	4--5	1293	51
SDC4	RefSeq	NM_011521.2	NP_035651.1	4--5	469	55
MMP2	RefSeq	NM_008610.2	NP_032636.1	2--3	674	62
MMP9	RefSeq	NM_013599.3	NP_038627.1	12--13	2098	76
MMP14	RefSeq	NM_008608.3	NP_032634.3	1--2	376	61
GAPDH	RefSeq	NM_001289726.1	NP_001276655.1	2--3	117	107

Table S3: List of primer probes used in this study
