# Supplementary material for: Blocking matrix metalloproteinase-mediated syndecan-4 shedding restores the endothelial glycocalyx and glomerular filtration barrier function in early diabetic kidney disease
Source: Kidney Int. 2020 May;97(5):951–65. doi: 10.1016/j.kint.2019.09.035 (PMC7184681; doi:10.1016/j.kint.2019.09.035)
Supplement: Table S4 — Absolute values of albumin, creatinine, and albumin creatinine ratio from 3 independent experiments carried out on diabetic (Dia) + MMPI or vehicle (Veh) mice. [file mmc9.pptx]

## Slide 1
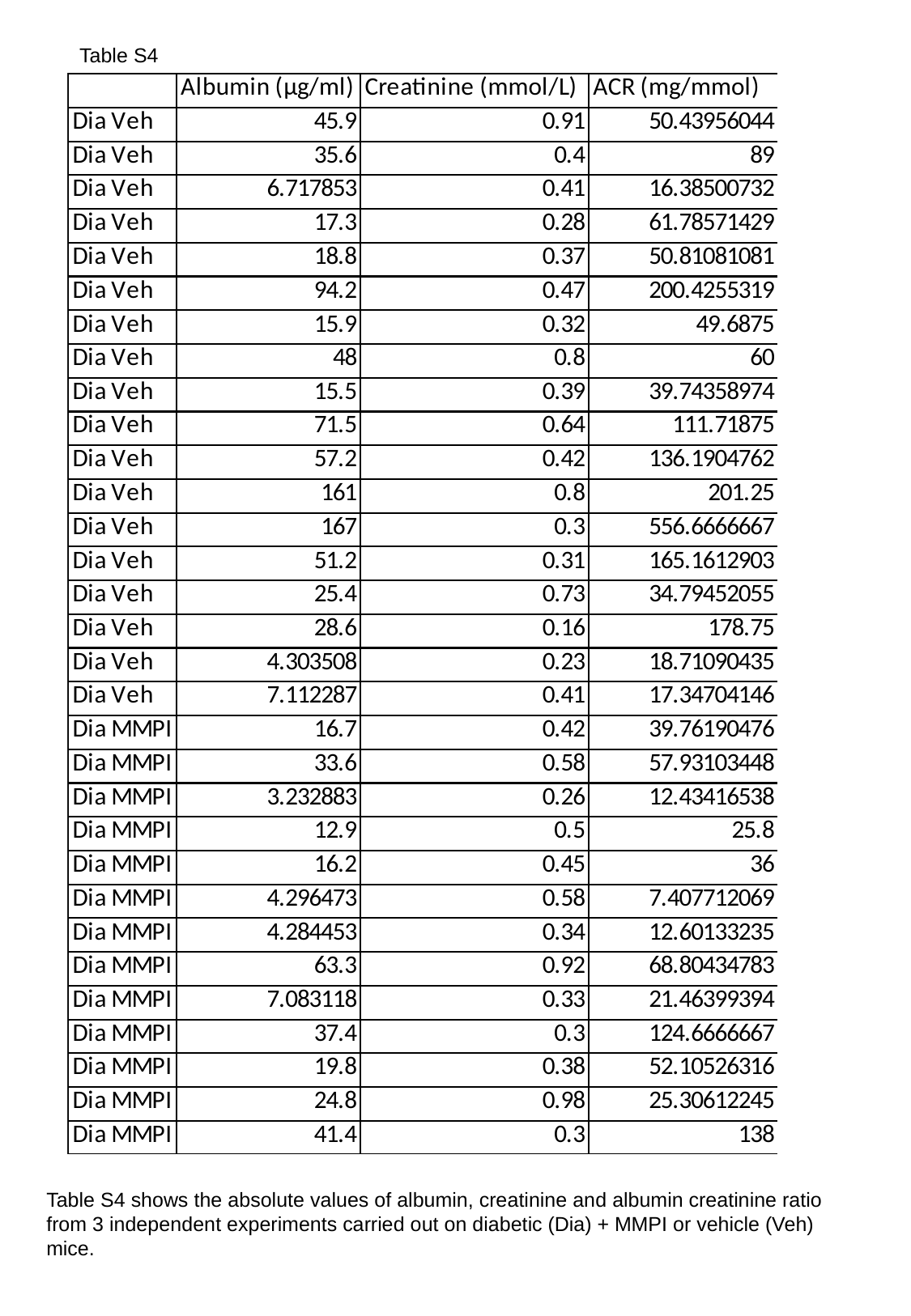

Table S4
Table S4 shows the absolute values of albumin, creatinine and albumin creatinine ratio from 3 independent experiments carried out on diabetic (Dia) + MMPI or vehicle (Veh) mice.
